# N^6^-methyladenosine reader protein YTHDC1 regulates influenza A virus NS segment splicing and replication

**DOI:** 10.1371/journal.ppat.1011305

**Published:** 2023-04-13

**Authors:** Yinxing Zhu, Ruifang Wang, Jiahui Zou, Shan Tian, Luyao Yu, Yuanbao Zhou, Ying Ran, Meilin Jin, Huanchun Chen, Hongbo Zhou

**Affiliations:** 1 State Key Laboratory of Agricultural Microbiology, College of Veterinary Medicine, Huazhong Agricultural University, Wuhan, China; 2 Key Laboratory of Preventive Veterinary Medicine in Hubei Province, Cooperative Innovation Center for Sustainable Pig Production, Wuhan, China; 3 Hubei Hongshan Laboratory, Wuhan, China; Indiana University Bloomington, UNITED STATES

## Abstract

N^6^-methyladenosine (m^6^A) modification on viral RNAs has a profound impact on infectivity. m^6^A is also a highly pervasive modification for influenza viral RNAs. However, its role in virus mRNA splicing is largely unknown. Here, we identify the m^6^A reader protein YTHDC1 as a host factor that associates with influenza A virus NS1 protein and modulates viral mRNA splicing. YTHDC1 levels are enhanced by IAV infection. We demonstrate that YTHDC1 inhibits NS splicing by binding to an NS 3′ splicing site and promotes IAV replication and pathogenicity *in vitro* and *in vivo*. Our results provide a mechanistic understanding of IAV-host interactions, a potential therapeutic target for blocking influenza virus infection, and a new avenue for the development of attenuated vaccines.

## Author summary

Influenza A virus (IAV) causes significant morbidity and mortality in humans and animals worldwide. Although we have learned a lot about the influenza virus over the past several decades, the detailed interaction patterns between host proteins and viral proteins that lead to infection remain underexplored. Here, we determined that YTHDC1, an m^6^A reader, was required for influenza virus replication. Our research suggests that YTHDC1 inhibits NS splicing by binding to an NS 3′ splicing site and promotes IAV replication and pathogenicity *in vitro* and *in vivo*. Our results provide an uncovered new mechanism into the biology and pathogenicity of the influenza A virus.

## Introduction

Influenza A virus (IAV) belongs to the Orthomyxoviridae family of RNA viruses and infects mammals and birds. Pathogenic strains of IAV cause high mortality in humans, which results in as many as annual death of 650,000 worldwide [1]. In addition to epidemics, pandemics sporadically occur when a novel IAV jumps to humans from an animal reservoir [2].

IAV is an enveloped virus with a genome comprised of eight single-stranded, negative-sense RNA segments that encode as many as 18 proteins [3]. Three segments (PB2, M, and NS) undergo alternative splicing [4,5]. The unspliced NS segment expresses non-structural protein 1 (NS1) and the spliced NS segment expresses nuclear export protein (NEP). NS1 is one of the earliest proteins expressed in virus infection and has important functions in the inhibition of host antiviral gene expression and virus replication [6,7]. NEP is essential for virus transcription, replication, and vRNP (viral ribonucleoprotein) nuclear export [7]. However, it is largely unknown how NEP/NS1 expression is regulated during virus replication. Several reports suggest that NS1 protein may be involved in the regulation of the NS mRNA splicing, but other studies argue against a role for NS1 in this process [6,8–11].

N^6^-methyladenosine (m^6^A) modifications are written by ‘writer’ proteins including methyltransferase-like (METTL) enzymes METTL3 and METTL14, and the cofactors Wilms tumor 1-associated protein (WTAP) and KIAA1429 complexes. m^6^A residues are detected by ‘reader’ YTH family proteins containing YTH-domain family 1 (YTHDF1), YTHDF2, YTHDF3, YTHDC1, and YTHDC2 proteins that bind to m^6^A through their C-terminal YTH domain [12]. m^6^A modification has been proposed to regulate mRNA function at multiple steps, including splicing, stability, translation, and secondary structure [13]. A previous study shows that m^6^A enhances IAV gene expression and replication but the mechanism remains unclear [14] and whether m^6^A regulates IAV mRNA splicing remains unknown.

In the present study, we apply a proteomic approach to unbiasedly identify host proteins that interact with the NS1 protein encoded by the IAV. Our results show that NS1 interacts with an m^6^A reader protein YTHDC1. YTHDC1 inhibits NS splicing by binding to NS 3′ splicing site, causing an enhancement of IAV replication and pathogenicity *in vitro* and *in vivo*. Our findings provide a potential target for blocking influenza virus infection, as well as a reference for the development of attenuated vaccines.

## Results

### NS1 interacts with YTHDC1

**Fig 1:**
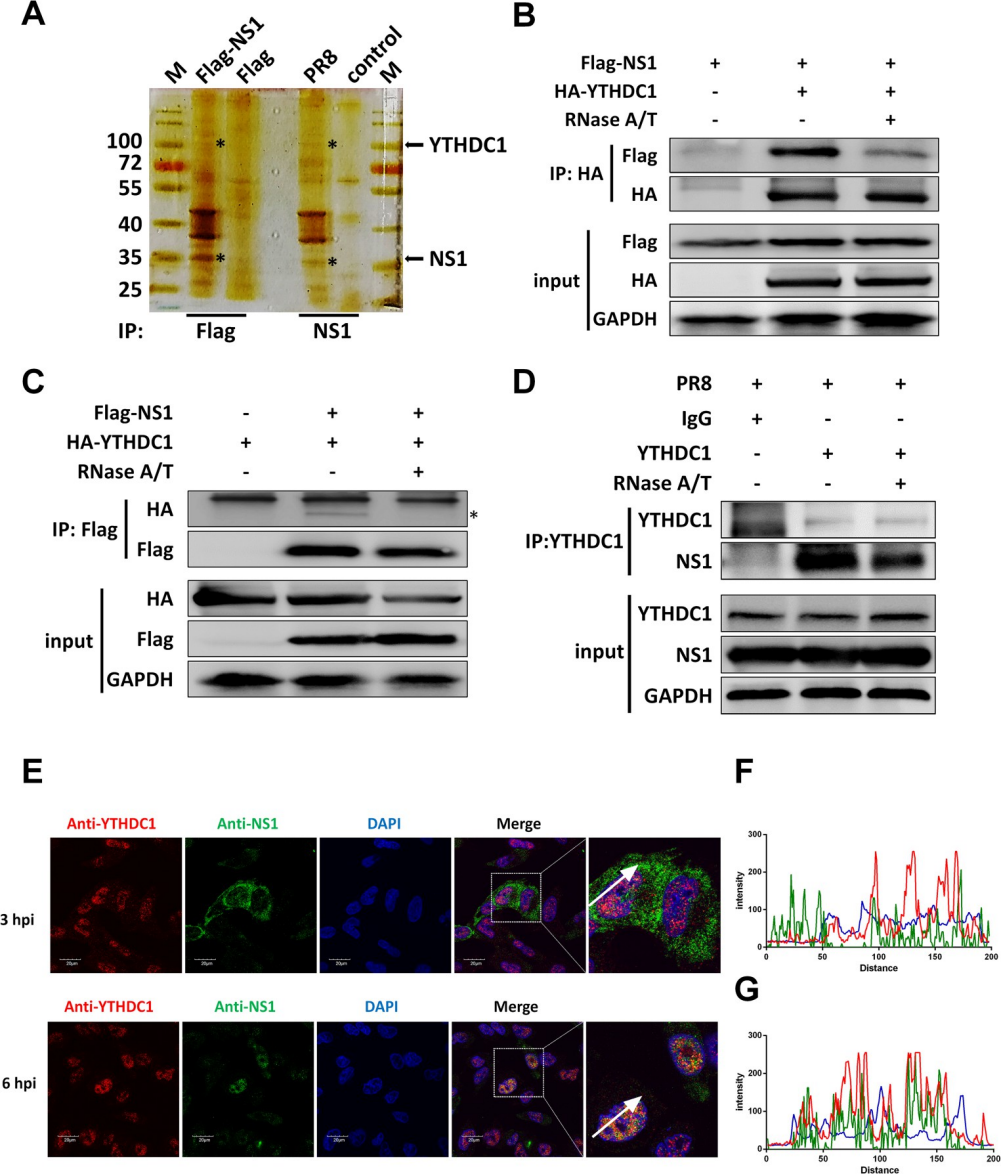
NS1 associates with YTHDC1. (A) HEK293T cells were infected with PR8 virus at a multiplicity of infection (MOI) of 0.01 or transfected with Flag-NS1 and vector for 24 hours, cell lysates were immunoprecipitated with an anti-Flag or anti-NS1 antibody and then analyzed by silver stain. (B-C) HEK293T cells were transfected with the indicated plasmids for 24 h. The cell lysates were treated with or without 100U RNase A/T at 37°C for 30 min. CoIP assay was performed using an anti-HA (B) and anti-Flag (C) antibody and analyzed by western blotting. (D) A549 cells were infected with the PR8 virus at an MOI of 0.01 for 24 hours. The cell lysates were treated with or without 100U RNase A/T at 37°C for 30 min. CoIP assay was performed using an anti-YTHDC1 antibody. Input and IP complexes (NS1 and YTHDC1) were analyzed by western blotting. (E-G) A549 cells were infected with the influenza virus at MOI of 5. Cells were fixed and analyzed for the colocalization of YTHDC1 with NS1 after 3 and 6 hours post infection. Scale bar, 20 μm. (F and G) The normalized fluorescence of YTHDC1 and NS1 along the white arrowheads shows overlapping peaks.

It has been proposed that NS1 can regulate the mRNA splicing of IAV and host through binding to RNA and interacting with host factors [6,15,16]. To investigate the new host factors associated with NS1, we used the NS1 affinity purification method to identify host factors that interact with NS1. HEK293T cells were transfected with Flag-tagged NS1 or infected with influenza virus, and we immunoprecipitated the proteins that interacted with NS1 with the anti-Flag or anti-NS1 beads and analyzed the proteins by mass spectrometry coupled with bioinformatics methods (Figs 1A and S1A). 763 specific proteins were identified in the Flag-NS1 transfection group compared to the vector transfection group, and 344 specific proteins were identified in the infected group compared to the mock infection group (S1B Fig). There was an overlap of 150 proteins in the transfection and infection group, which were summarized in S1 Table. We found that P85B, SNF2L, P85A, and RALY potentially interact with NS1, which has been confirmed by previous studies [17–19]. A host protein named YTHDC1 was one of the candidate proteins that may potentially interact with NS1. The interaction between NS1 and YTHDC1 was confirmed by coimmunoprecipitation (CoIP) assays. Ectopically expressed Flag-tagged NS1 co-precipitated with HA-tagged YTHDC1 (Fig 1B). The reverse CoIP experiment showed that the YTHDC1 protein specifically interacted with NS1 (Fig 1C). We further investigated whether endogenous YTHDC1 interacts to viral NS1, and results showed that the YTHDC1 binds with NS1 protein in IAV (PR8/H1N1)-infected A549 cells (Fig 1D). To determine whether this interaction depends on RNA, we detected the interaction by an RNA digestion assay. Results showed that the NS1 and YTHDC1 interaction was at least partly RNA dependent (Fig 1B and 1D, line 3). Moreover, the immunofluorescence assay reflected that NS1 proteins colocalized with YTHDC1 in the nucleus of virus-infected A549 cells (Fig 1E) and co-transfected A549 cells (S1C and S1D Fig). These results suggest that NS1 interacts with YTHDC1 in the nucleus, partly dependent on RNA.

### IAV infection enhances YTHDC1 expression

Even though some writer and reader proteins involved in m^6^A modification have been reported to regulate the m^6^A of IAV transcripts to promote IAV replication [14], whether IAV infection can in turn affect the level of these proteins remains unclear. Firstly, we detected the protein level of YTHDC1 in IAV-infected A549 cells. Results showed that IAV infection promoted the YTHDC1 protein expression but not the mRNA level at both the early and late stages of virus-infected cells (S2A and S2B Fig). Our indirect immunofluorescence assay further confirmed the increase of YTHDC1 after cells were infected with IAV (S2C Fig). These results reveal that IAV positively regulates YTHDC1 expression.

### YTHDC1 promotes IAV replication

**Fig 2:**
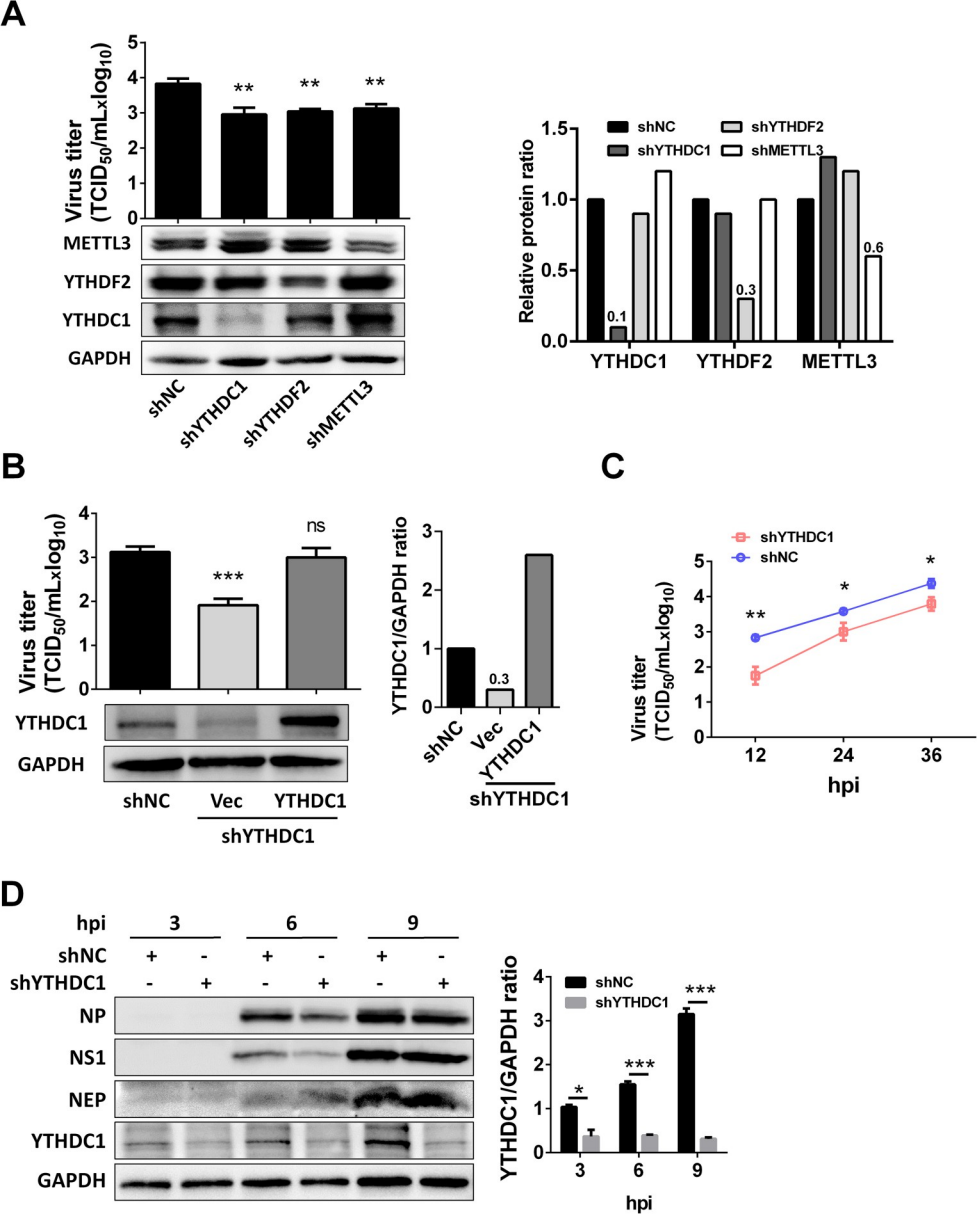
YTHDC1 promotes PR8 virus replication. (A) METTL3, YTHDF2, and YTHDC1 were knocked down in A549 cells by shRNA, and then infected with the PR8 virus. Virus titer and protein expression were detected by TCID_50_ and western blotting at 24 hpi (left). Each protein band was quantified by ImageJ and normalized to GAPDH levels (right). Data are presented as the average of three experiments and error bars indicate the standard error of the mean (SEM) (one-way ANOVA; **, P<0.01). (B) WT and YTHDC1 knockdown A549 cells were transfected with 0.5 μg YTHDC1 for 24 hours, vector as a control, and then infected with PR8 virus (MOI = 0.01) for 24 h. Virus titer was detected by TCID_50_ assay (left). Each protein band was quantified by ImageJ and normalized to GAPDH levels (right). Data are presented as the average of three experiments and error bars indicate the standard error of the mean (SEM) (Student t-test; ns, not significant; ***, P<0.001). (C) A549 cells were infected with the PR8 virus at an MOI of 0.01. Cell culture supernatants were collected at 12, 24, and 36 hpi. Virus titers were determined by TCID_50_ assay on MDCK cells. Data are presented as the average of three experiments and error bars indicate the standard error of the mean (SEM) (two-way ANOVA test; * P<0.05, **, P<0.01). (D) YTHDC1 knockdown (shYTHDC1) or negative control (shNC) knockdown A549 cells were infected with the PR8 virus at an MOI of 5. Cell lysates were subjected to western blotting analysis at 3, 6, and 9 hpi. GAPDH was used as a loading control. Each protein band was quantified by ImageJ and normalized to GAPDH levels. The YTHDC1/GADPH protein ratio was calculated. Data are presented as the average of three experiments and error bars indicate the standard error of the mean (SEM) (one-way ANOVA; ns, not significant; *, P<0.05; ***, P<0.001).

YTH proteins bind to m^6^A modifications on single-stranded RNA via a conserved domain located at their C terminus [20]. Influenza A virus HA, NA, M, and NS have m^6^A modification [14]. It has been proposed that the m^6^A reader protein YTHDF2 and writer protein METTL3 are able to support the IAV replication [14], we next sought to investigate whether the m^6^A reader protein YTHDC1 was able to regulate IAV replication. The YTHDC1-, YTHDF2-, and METTL3-knockdown A549 cells were established by short hairpin RNA (shRNA) and knockdown YTHDC1 did not change cell viability (S3A Fig). Notably, the knockdown of YTHDF2 and METTL3 proteins resulted in a decrease in viral replication at 24 hours post infection (hpi) (Fig 2A), which were consistent with a previous study [14]. Moreover, knockdown of YTHDC1 markedly inhibited viral replication, whereas overexpression of YHTDC1 protein in YTHDC1 knockdown cells reversed the virus replication, which showed a similar level to the control knockdown cells (Fig 2B). We further revealed that knockdown of YTHDC1 inhibited IAV replication at 12, 24, and 36 hpi (Fig 2C). To explore whether YTHDC1 affects IAV replication at an early stage of IAV infection, we monitored viral protein levels at 3, 6, and 9 hpi in IAV infected A549 cells. Knockdown of YTHDC1 decreased the protein expression levels of NP and NS1 (Fig 2D), and mRNA, vRNA, and cRNA levels of NP also declined (S3B–S3D Fig), compared with those in IAV-infected control knockdown cells. These results suggest that the m^6^A reader protein YTHDC1 supports influenza virus replication.

### YTHDC1 binds to the NS 3′ splicing site and regulates NS segment splicing

**Fig 3:**
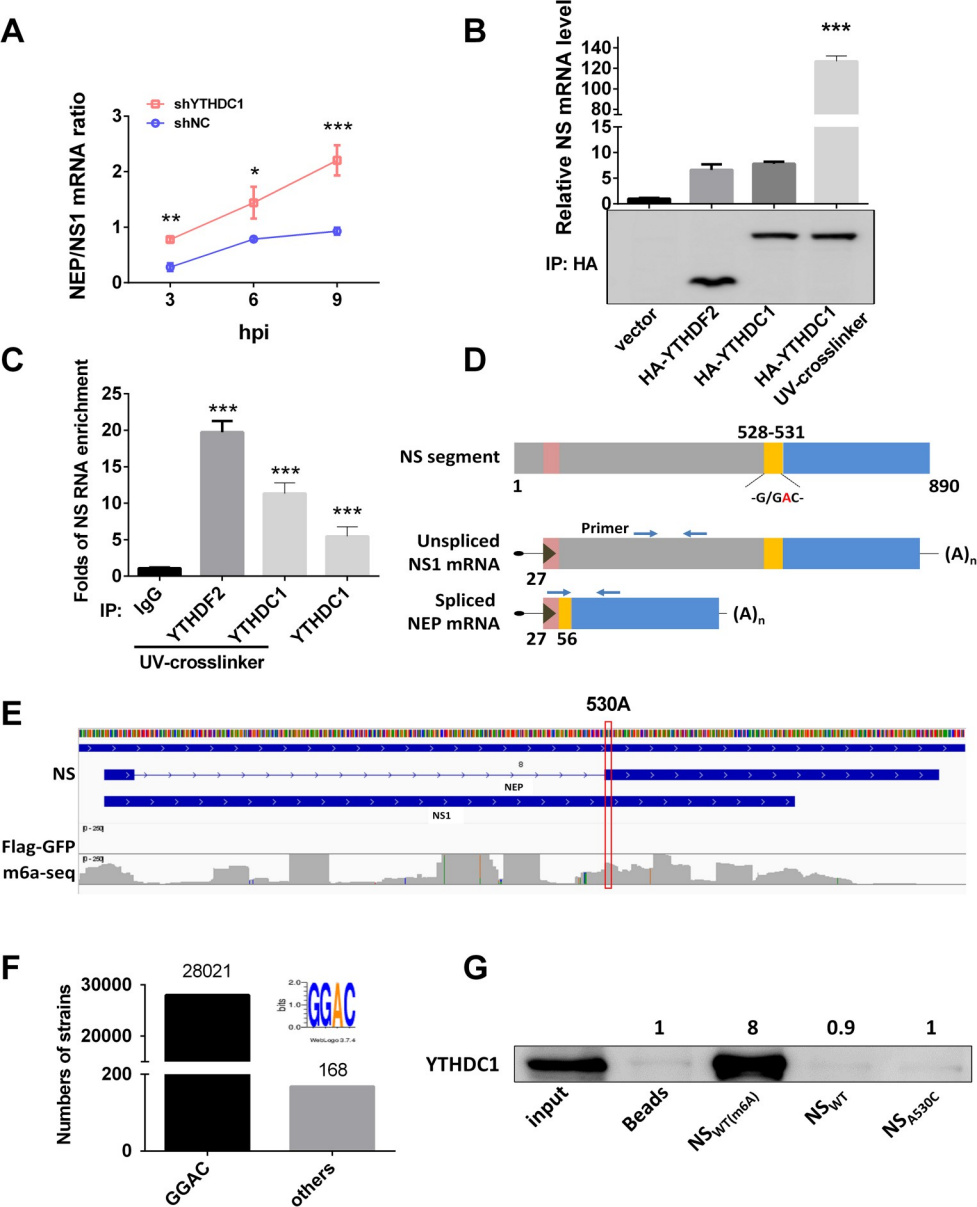
NS1 inhibits NS segment splicing by YTHDC1. (A) YTHDC1 knockdown (shYTHDC1) or negative control (shNC) knockdown A549 cells were infected with the PR8 virus at an MOI of 5. The ratios of NEP to NS1 mRNA were calculated at indicated time points with the specific primers. Data are presented as the average of three experiments and error bars indicate the standard error of the mean (SEM) (two-way ANOVA test; * P<0.05, ** P<0.01; ***, P<0.001). (B) HEK293T cells were transfected with indicated plasmids for 24 h and then infected with the PR8 virus at an MOI of 1 for 12 h. RIPs and RT-qPCR were performed using specific primers detecting viral NS mRNA. Fold enrichment of mRNA was calculated. Data are presented as the average of three experiments and error bars indicate the standard error of the mean (SEM) (one-way ANOVA; ***, P<0.001). (C) A549 cells were infected with the PR8 virus at an MOI of 1 for 12 h. RIPs and RT-qPCR were performed using specific primers detecting viral NS mRNA. Fold enrichment of mRNA was calculated. Data are presented as the average of three experiments and error bars indicate the standard error of the mean (SEM) (one-way ANOVA; ***, P<0.001). (D) NS1 mRNA and its alternatively spliced product were depicted, and the arrowheads show primer positions for detection of various. G/GAC (528–531) is the YTHDC1-binding site and ‘/’ is the 3′ splicing site in the NS segment. (E) A view of the NS segment of IAV-PR8. PA-m^6^A-seq has a y-axis of 0–250 reads[14]. The 530A site is marked in the red box. (F) Analysis of GGAC motif in NS 528–531 segment of IAVs, total of 28189 strains were analyzed. (G) Pulldown YTHDC1 proteins using 20 bp NS biotin-probes of PR8: probes were immunoprecipitated with the cell lysis. And the bead eluate was analyzed by western blotting.

Given the abnormal ratio of NEP/NS1 protein expression (Fig 2D), we suspected that YTHDC1 is involved in the splicing of the NS segment. To test this, we knocked down YTHDC1 and infected A549 cells with IAV at an MOI of 5. Results showed that knockdown of YTHDC1 remarkedly inhibited the NS1 mRNA level (S3E Fig) but not the NEP mRNA level (S3F Fig), which promoted the NEP/NS1 mRNA ratio at 3, 6, and 9 hpi (Fig 3A), suggesting that YTHDC1 affected NEP splicing. However, the expression of YTHDC1 did not affect PB2 splicing (S3G Fig) and knockdown YTHDC1 did not change NS mRNA stability (S3H Fig). Considering YTHDC1 is an m^6^A reader protein that binds to mRNA, we next determined whether YTHDC1 targets the NS mRNA, resulting in the decrease of NS mRNA splicing under virus infection. RNA immunoprecipitation (RIP) and reverse transcription-quantitative polymerase chain reaction (RT-qPCR) analysis showed that NS mRNA was enriched more than 120-fold by precipitation of HA-YTHDC1 in HA-YTHDC1-transfected cells compared with control vector-transfected cells (Fig 3B) and ten-fold by endogenous YTHDC1 compared to that by IgG (Fig 3C). The favored methylation sequence for m^6^A modification in mRNA is RRACU or at least RAC [21]. Previous studies have shown that YTHDC1 carries a YTH domain, which can bind to a GG(m^6^A)C motif [20,22]. The short consensus at 5′ and 3′ splicing sites (5′AG/GT——NAG/GNN3′; no strict conservation) in higher eukaryotic RNA splicing seem to rule out any direct marking of splicing sites by m^6^A modification [23]. However, we observed that the NS segment of the PR8/H1N1 virus carries a G/GAC (528–531) motif which is overlapped with the 3′ splicing site (CAG/GAC) (Fig 3D). To explore whether this motif was under m^6^A modification, we analyzed the m^6^A-seq data from reference [14], and results showed that the motif GGA(530)C was an m^6^A modification site (Fig 3E) and overlap with the splicing site. This motif is very conservative in IAVs (28021/28189 strains) (Fig 3F). We next performed an RNA pull-down assay by using 20-base pair (bp) biotin-labeled probes with m^6^A modification GG(m^6^A)C motif (NS_WT (m6A)_) or no modification motif (NS_WT_) or a mutated motif (NS_A530C_). Results showed that YTHDC1 is preferably bound to the m^6^A modified wild type NS (NS_WT (m6A)_), in comparison with the unmodified NS and A530C mutated NS (NS_A530C_) (Fig 3G). Taken together, our results demonstrate that the GG(m^6^A)C motif at the 3′ splicing site of NS mRNAs is required for its association with YTHDC1 and NS segment splicing.

### NS1 regulates NS segment splicing through interaction with YTHDC1

**Fig 4:**
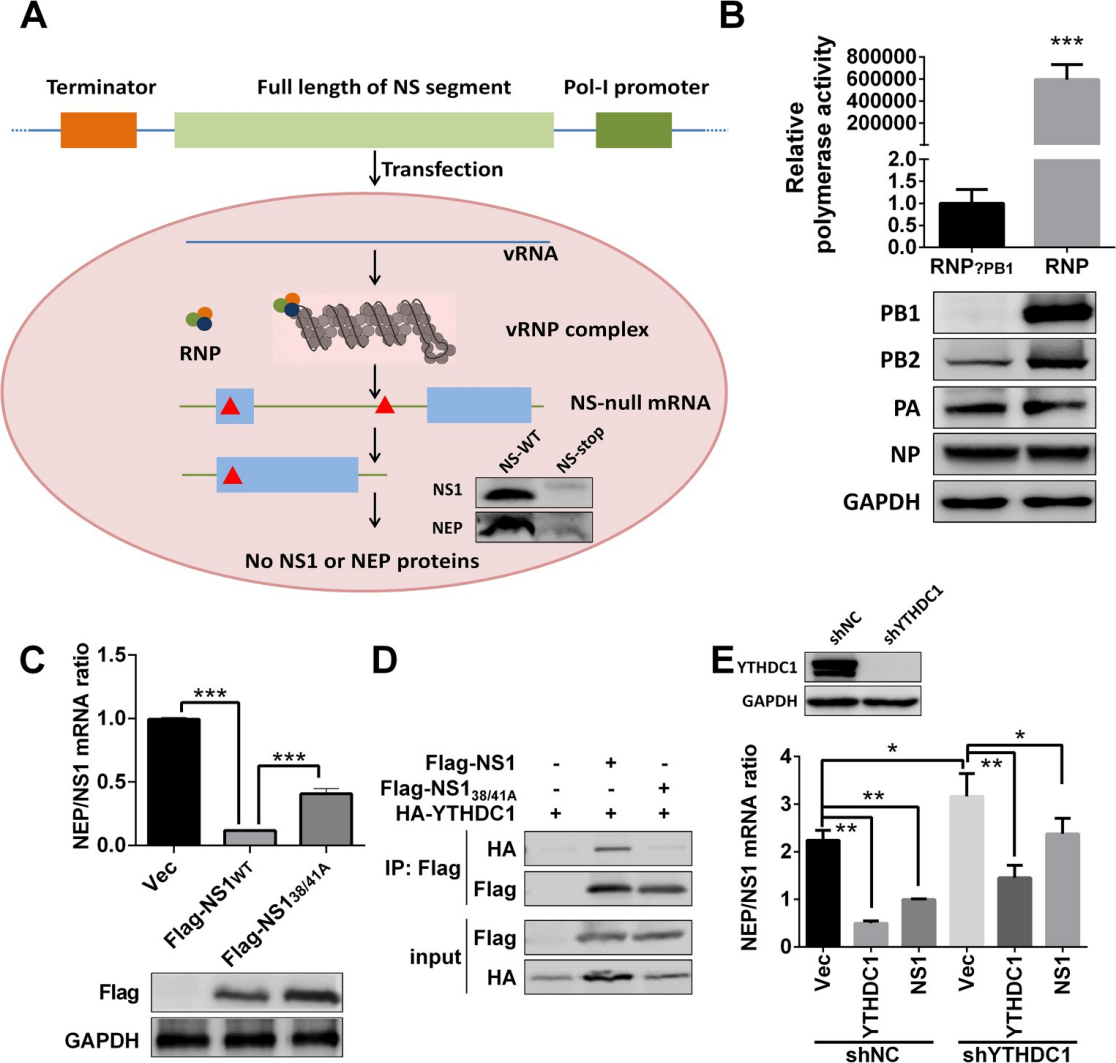
NS1 regulates NS segment splicing through YTHDC1. (A) Schematic illustration of NS-null minigenome system and detection of specific NS mRNAs. Construction of the pHI NS-null plasmid, which contains multiple stop codons (red triangle). Primers used for NS-null mRNA detection are shown as blue arrowheads. 3P (PA, PB1, PB2). NS-null (two stop codons were mutated at positions 45 and 456, with transcription but not translation). (B) HEK293T cells were transfected with the indicated viral RNP reconstitution plasmids (pCDNA-3.1-PB1, -PB2, -PA, and -NP, pPolI-Luc, and Renilla); polymerase activity was measured at 24 h post transfection. Data are presented as the average of three experiments and error bars indicate the standard error of the mean (SEM) (Student t-test; ***, P<0.001). (C) HEK293T cells were transfected with the minigenome system and Flag-NS1 or Flag-NS1_38/41A_ for 24 h, total RNAs were extracted and quantified by RT-qPCR with specific primers, and NEP/NS1 mRNA was calculated. Data are presented as the average of three experiments and error bars indicate the standard error of the mean (SEM) (one-way ANOVA; ***, P<0.001). (D) HEK293T cells were transfected with the indicated plasmids for 24 h. CoIP assay was performed using an anti-Flag antibody and analyzed by western blotting. (E) YTHDC1 was knocked down by shRNA on HEK293T cells. And indicated plasmids were transfected with the minigenome system, and related NEP/NS1 ratios were detected at 24 hours post transfection. Data are presented as the average of three experiments and error bars indicate the standard error of the mean (SEM) (one-way ANOVA; *, P<0.05; **, P<0.01).

NS1 protein has been reported to regulate IAV NS segment splicing by interacting with host factors [8]. Given that NS1 was able to bind to YTHDC1 protein in our above results (Fig 1B–1D), next we sought to explore whether NS1 regulates NS segment splicing via the interaction of NS1 protein with YTHDC1. An IAV polymerase cell-based transcription system was established to assess the splicing efficiencies without expressing NS1 or NEP proteins (Fig 4A). In this system, NS-vRNA was first generated by a pol-I-driven plasmid and then NS mRNA is transcribed from vRNA by viral ribonucleoprotein complex (vRNP, viral polymerase 3P (PA, PB1, PB2)+NP) (Fig 4A). We confirmed that the minigenome system works well by a dual luciferase assay (Fig 4B). Intriguingly, by using this minigenome system, our results showed that NS1 protein inhibited NS segment splicing, NS1 R38A and K41A (NS_38/41A_) double mutations attenuated its ability to inhibit NS segment splicing (Fig 4C). NS1_38/41A_ mutations inhibited the interaction of YTHDC1 and NS1 (Fig 4D), and previous study shows that the amino acid 38 and 41 double mutations in NS1 protein abolish its binding ability to viral mRNA [24], suggesting that NS1 inhibits NS segment splicing dependent on influenza NS1:RNA. Knockdown of YTHDC1 promoted NS splicing and restoring YTHDC1 inhibited NS splicing (Fig 4E). NS1 inhibited the splicing of the NS segment, but NS1 slightly inhibit NS segment splicing in YTHDC1 knockdown cells (Fig 4E). Taken together, these results suggest that NS1 inhibited the splicing of NS mRNA by interacting with YTHDC1.

### NS A530C single mutation alters virus replication and NS splicing

**Fig 5:**
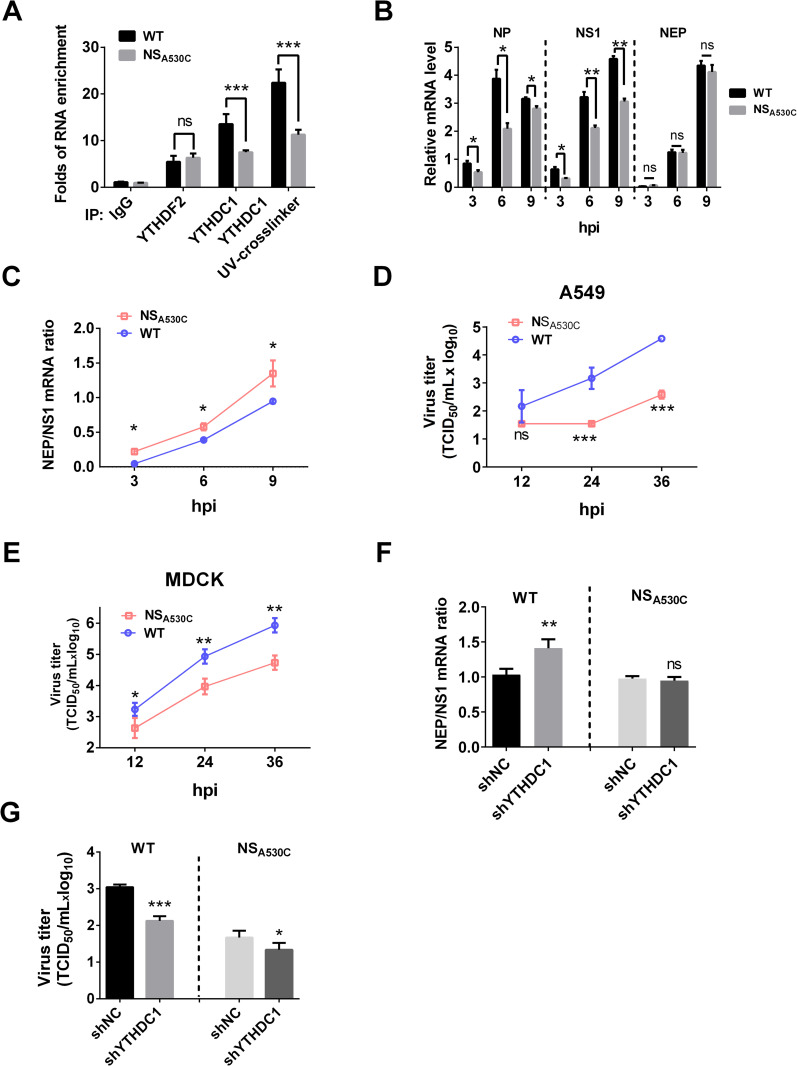
NS_A530C_ mutation altered virus splicing and replication. (A)A549 cells were infected with the wild type or mutated PR8 virus at an MOI of 1 for 12 h, and the mRNAs were purified with either the control IgG or the YTHDC1 antibody. The purified RNAs were then analyzed by specific RT-qPCR assay. Data are presented as the average of three experiments and error bars indicate the standard error of the mean (SEM) (one-way ANOVA; ns, not significant; ***, P<0.001). (B) A549 cells were infected with either the wild type or mutant of the PR8 virus at an MOI 5 for the indicated periods. The total RNA was isolated and analyzed by specific RT-qPCR targeting NP, NEP and NS1 mRNA, which normalized to GAPDH mRNA. Data are presented as the average of three experiments and error bars indicate the standard error of the mean (SEM) (two-way ANOVA; ns, not significant; *, P<0.05; **, P<0.01; ***, P<0.001). (C) A549 cells were infected with the indicated virus at an MOI of 5, the total RNA was isolated and analyzed by specific RT-qPCR targeting NEP and NS1 mRNA. Ratios of NEP/NS1 mRNA are presented. Data are presented as the average of three experiments and error bars indicate the standard error of the mean (SEM) (two-way ANOVA; *, P<0.05). (D-E) (D)A549 cells and (E) MDCK cells were infected with either the wild type or mutant of the PR8 virus at an MOI 0.01 for the indicated periods. The growth curves were determined by TCID_50_ analysis on MDCK cells. Data are presented as the average of three experiments and error bars indicate the standard error of the mean (SEM) (two-way ANOVA; ns, not significant; **, P<0.01; ***, P<0.001). (F) A549 cells were infected with the wild type (WT) and mutant (NS_A530C_) PR8 virus at an MOI of 5. The total RNAs were measured by RT-qPCR and the NEP/NS1 mRNA ratios were calculated. Data are presented as the average of three experiments and error bars indicate the standard error of the mean (SEM) (one-way ANOVA; ns, not significant; **, P<0.01; ***, P<0.001). (G) A549 cells were infected with the wild type (WT) and mutant (NS_A530C_) PR8 virus at an MOI of 0.01. The virus titers were detected at 24 hpi. Data are presented as the average of three experiments and error bars indicate the standard error of the mean (SEM) (one-way ANOVA; ns, not significant; **, P<0.01; ***, P<0.001).

To investigate the effect of GG(m^6^A)C at the 3′ splicing site of NS on viral mRNA splicing and viral replication, we generated a mutant PR8 virus with NS_A530C_ mutation. The synonymous mutation did not change NS1 amino acids. We determined whether the NS_A530C_ mutation altered the binding efficiency to YTHDC1 under virus infection. Results of RIP assay showed that the NS_WT_ enriched by YTHDC1 was two-fold higher than that of NS_A530C_ (Fig 5A). To analyze whether the identified NS change affects its splicing, A549 cells were infected with IAV, and the NEP/NS1 mRNA levels were determined at different time points using RT-qPCR assay. Results showed that the NP and NS1 mRNA were obviously increased in wild type (WT) virus-infected A549 cells compared with NS_A530C_ virus-infected cells, and NEP/NS1 mRNA ratios were decreased (Fig 5B and 5C). To assess the impact of NS_A530C_ mutation on viral replication, we compared the growth kinetics of WT and mutated viruses in different cell lines. Results showed that the single mutation in NS (NS_A530C_) notably inhibited virus replication on A549 cells, MDCK cells, and MAVS KO MDCK cells (Figs 5D, 5E and S4), suggesting that NS_A530C_ attenuation is not dependent on interferon.

To further confirm that YTHDC1 regulates viral NS splicing to affect viral replication, control, and YTHDC1 knockdown A549 cells were infected with wild type or NS_A530C_ mutated virus to determine NS mRNA splicing. Results showed that the knockdown of YTHDC1 did not change the NS splicing of the mutated virus (Fig 5F), but inhibited virus replication (Fig 5G), suggesting that YTHDC1 regulates IAV replication by regulating NS splicing. These results indicate that restoring the m^6^A modification in NS-530 through a single mutation in NS_A530C_ reduces its binding with YTHDC1 and enhances its mRNA splicing, resulting in increasing the ratio of NEP/NS1 and subsequently inhibiting virus replication.

### m^6^A single mutation at NS_A530C_ alters virus pathogenicity in mice

**Fig 6:**
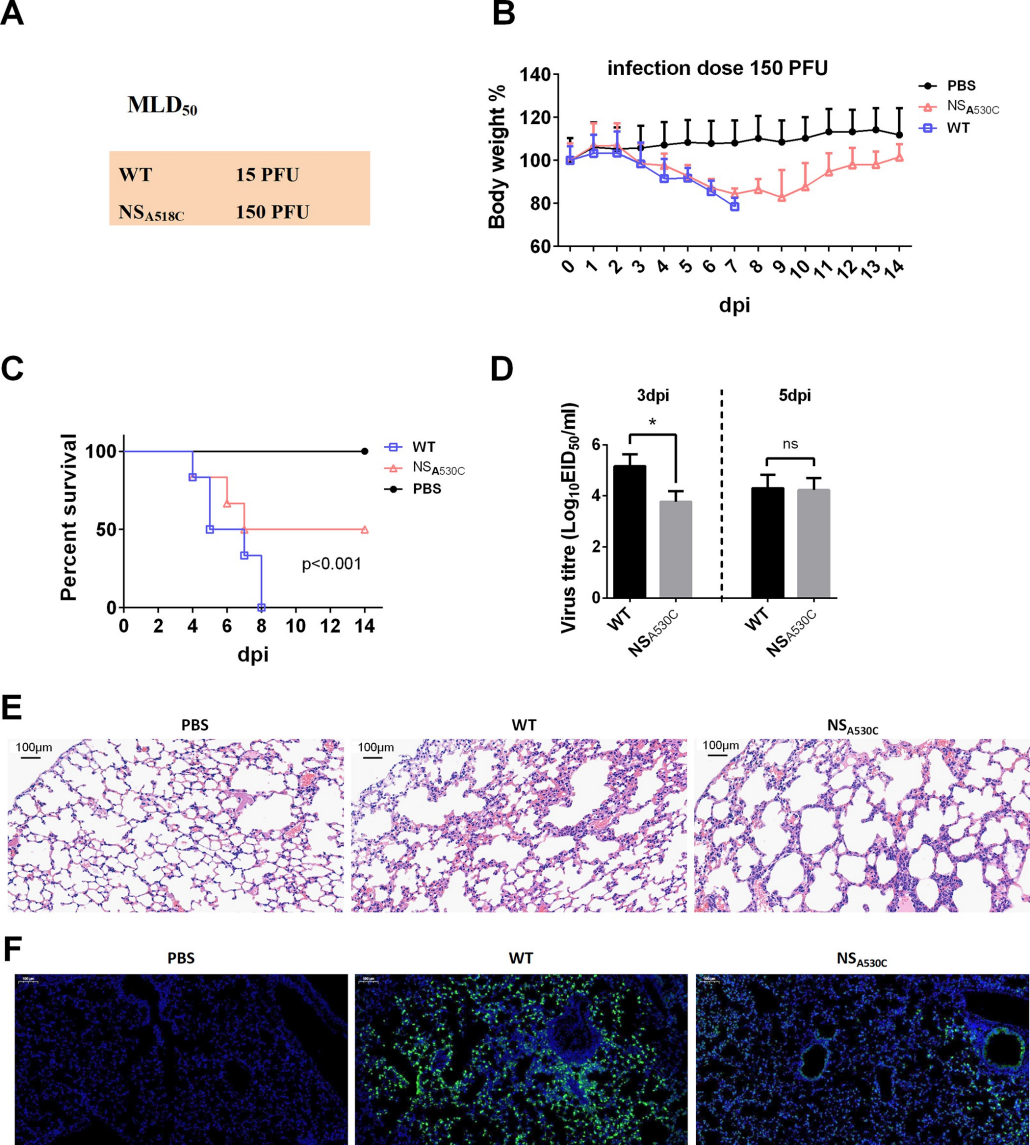
NS_A530C_ mutation inhibited virus virulence. (A) MLD_50_ of WT and mutant viruses. MLD_50_ was calculated by the Reed and Muench method. (B and C) Weight loss and mortality of mice infected with each indicted virus. The body weight of the WT and mutant groups was compared and statistically analyzed. Error bars represent means ± SEM (n = 10). Statistical analysis of (C) was performed by using Log-rank (Mantel Cox) test. (D) Virus titers in the lungs of infected mice (n = 3) at 3 days (left) and 5 days (right) post-infection. Error bars represent means ± SEM. Statistical analysis was performed by using the one-way ANOVA method (n = 3, ns, not significant; *, P<0.05). (E) Pathological lesions in the lungs of mice infected with the indicated virus at 3 days post-infection with hematoxylin and eosin (H&E) staining. Scale bars, 100 μm. (F) Immunofluorescent staining of lung sections of mice infected with the indicated virus at 3 dpi. The viral NP antigen was stained green, and the nucleus was stained blue. Scale bars, 1000 μm.

Given that the single mutation at position 530 in NS altered its mRNA splicing and viral replication in human cells, next we determined the pathogenicity of the mutated viruses in BALB/c mice. Results showed that NS_A530C_ virus infection significantly decreased pathogenicity compared with the WT virus infection, reflected by approximately 10-fold changes in the MLD_50_ (mouse median lethal dose) of WT and mutated viruses (Figs 6A and S5A–S5D). In addition, mice infected with the WT virus displayed severe weight loss and reached 100% mortality, in comparison with those infected with the same dose of the NS_A530C_ virus which caused 50% mortality (Fig 6B and 6C).

Virus titers in the lungs of mice infected with WT were more than 10-fold higher at 3 days post infection (dpi) than those detected in NS_A530C_ infected mice (Fig 6D). And there are no reversion mutations of NS_530_ at 5 dpi (S5E Fig). The lungs of mice infected with either WT or NS_A530C_ had moderate to severe bronchiolar necrosis, pulmonary edema, and inflammatory cell infiltrates, while the lung lymphoid tissue infiltration was restricted in NS_A530C_-infected mice at 3 dpi (Fig 6E). In addition, weaker NP antigen signals were detected in the lungs of mice infected with NS_A530C_ virus when compared with those infected with WT virus at 3 dpi (Fig 6F). These results indicate that mutation in the NS m^6^A site of IAV decreases viral pathogenicity in mammals.

## Discussion

There are more than 100 different chemical modifications in RNAs, one-third of which involve the addition of methyl groups to the RNA base. Among them, m^6^A modification is the most prevalent one [25]. m^6^A modification has been proposed to regulate mRNA function at multiple steps, including splicing, stability, translation, and secondary structure [13]. Though many studies have documented that deposition of m^6^A on viral transcripts can influence viral replication during IAV, Kaposi’s sarcoma-associated herpesvirus, simian virus 40, human immunodeficiency virus, Zika virus, hepatitis B and C virus, enterovirus 71, and respiratory syncytial virus infection [14,26–36], the regulation mechanisms of m^6^A modification in virus replication were not uniform. A previous study shows that IAV mRNA is enriched with m^6^A modification, and m^6^A enhances IAV gene expression and replication [14]. A recent study has reported that m^6^A modifications on adenovirus RNA are necessary for efficient splicing, which supports virus replication [37]. However, the mechanisms of whether and how m^6^A is involved in the regulation of IAV mRNA splicing remain unknown.

Influenza virus transcribes and replicates its viral genome using the viral polymerase complex, which relies on numerous host factors to support its RNA processing including mRNA splicing. mRNA splicing depends on the proper actions of splicing complex and regulators, which is thought to be one of the most complex cellular processes. Previous studies show that NS1-BP regulates IAV M segment splicing by interacting with hn-RNP K, and hn-RNP K enhances M splicing through binding to the M mRNA [16,38]. In addition, the splicing regulator SF2 interacts with IAV NS1 protein and binds to the exonic splicing enhancer of NS, thereby regulating NS segment splicing [8]. Our published study also has shown that the splicing regulator TRA2A binds to the intronic splicing silencer of different mRNA segment of avian and human IAV, then regulate its host adaptation [39]. However, whether other host factors except the splicing complex and regulators are involved in the IAV mRNA splicing remains unknown. Notably, our study first identified YTHDC1, an m^6^A reader protein, which binds to NS1 protein by both mass spectrometry and coimmunoprecipitation assay. Our study further demonstrates that YTHDC1 enhances IAV replication, as well as the m^6^A writer protein METTL3 and reader protein YTHDF2 as reported by a previous study [14]. Simultaneously, we show that the initial level of YTHDC1 is very low in human lung epithelial cells (Fig 2D), and IAV infection significantly increases the expression of YTHDC1 (Figs 2D and S2). However, the mechanism needs to be further studied.

A previous study has shown that YTHDC1 regulates mRNA splicing by interacting with the splicing enhancers SRSF3 and SRSF10 [40]. Interestingly, our study shows that YTHDC1 regulates NS segment splicing by directly binding to the 3′ splicing site of NS mRNA, resulting in promoting IAV replication and pathogenicity. Furthermore, we demonstrate that YTHDC1 recognizes the GG(m^6^A)C motif, a conserved motif in the NS segment of IAV, and 530-m^6^A in this motif is crucial for IAV splicing and replication.

Aquatic birds are the main reservoir of most of the influenza A viruses (IAVs) in nature [41]. The gene pool of IAVs in waterfowl provides all the genetic diversity required for the emergence of pandemic influenza viruses in poultry and humans. The spread of IAV from waterfowl to poultry and poultry to human is a gradual development process of IAV. As we know, the rapid evolution of IAVs in humans and other mammals has never stopped. We found that the GG(m^6^A)C motif is very conserved in the NS mRNA of IAV. Some H5, H7, and H9 IAVs isolated from waterfowl and poultry maintain NS 530-G/C, but all human-isolated subtypes are 530-A (S6 Fig and S2 Table), which suggests that the 530 site in NS might have been gradually completed its mutation to adapt to humans during the long-term evolution of avian IAV and further testing is required.

**Fig 7:**
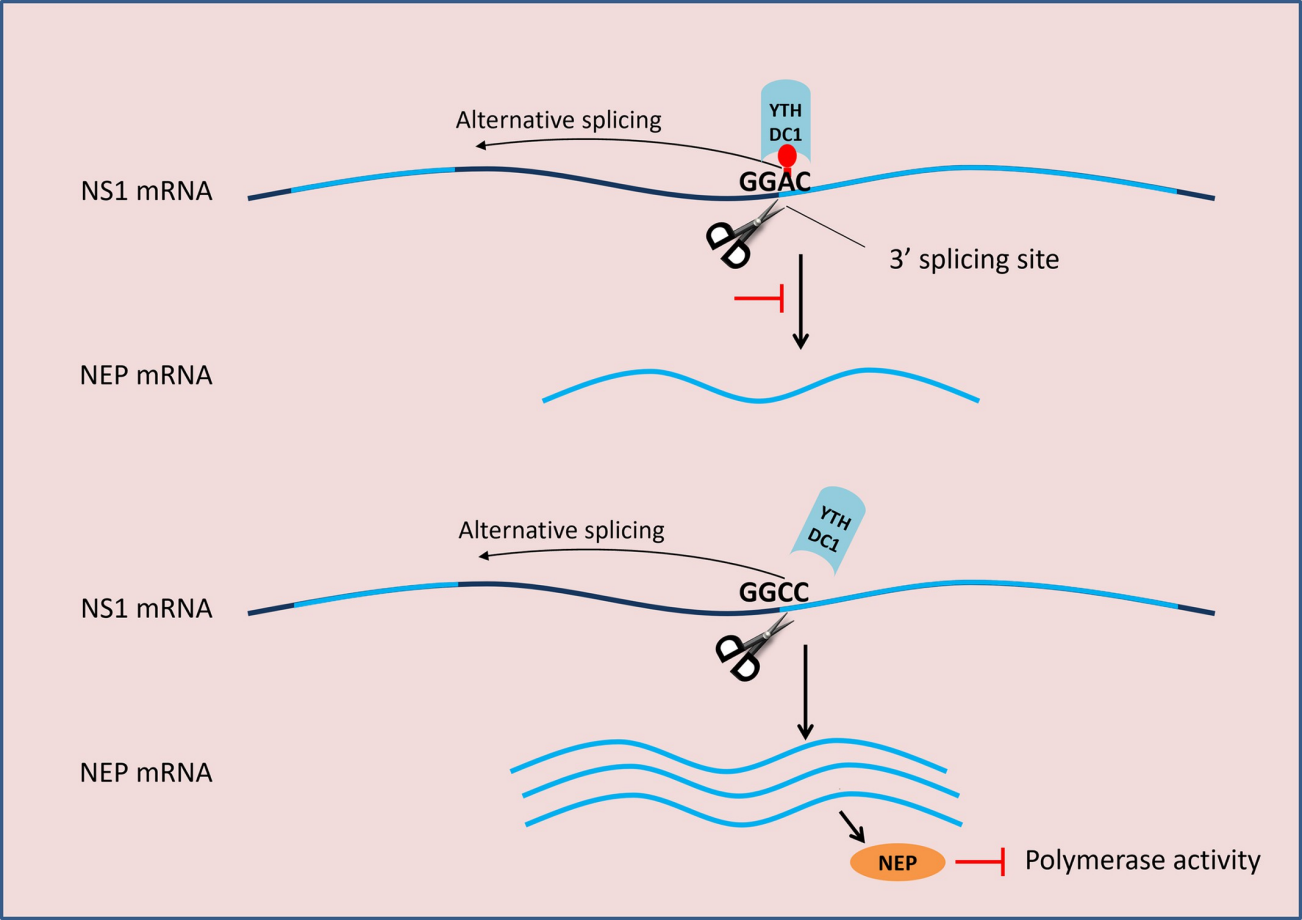
Working model for the regulation of NS mRNA splicing. NEP mRNA is a spliced form of NS1 mRNA. The splicing complex binds (scissors) to the 3’ splicing site of NS1 mRNA and regulates alternative splicing. YTHDC1 binds to the m^6^A modification site on the 3’ splice site and inhibits the splicing process. The A530C mutation results in reduced binding of NS mRNA by YTHDC1, which promotes NS1 mRNA splicing and produces more NEP, thereby inhibiting viral polymerase activity.

It has been documented that NS1 is involved in the regulation of IAV M segment splicing [10], but its role in NS segment splicing remains controversial [6,8,10,11]. In this present study, we find that NS1 inhibits NS segment splicing, which is consistent with a previous study [8]. It has known that NS1 expressed from unspliced NS is crucial for early viral replication [8], mRNA splicing, and nuclear export, and NEP expressed from spliced NS is essential for vRNP nuclear export [42]. However, more NEP will inhibit human IAV polymerase activity and virus replication at the early stage of IAV replication as reported in our previous study [39] and another previous study [43], indicating that less NEP favors human IAV replication (Fig 7). Binding of YTHDC1 to the NS segment 3′ splicing site may result in competition for the binding of splicing complexes. Consistent with our previous results, we show that YTHDC1 inhibits IAV NS segment splicing, leading to less NEP expression, and resulting in promoting IAV replication. But whether YTHDC1 affects M2/M1 splicing remains further study.

In summary, our study unveils a novel mechanism utilized by IAV to promote its replication and provides new insight into the pathogenicity of IAV. We identified an m^6^A reader protein, YTHDC1, interacts with NS1 protein. YTHDC1 binds to the NS 3′ splicing site of IAV, resulting in the inhibition of NS segment splicing, thereby benefiting IAV replication and pathogenicity. These findings provide a potential target for blocking influenza virus infection, as well as a reference for the development of attenuated vaccines.

## Materials and methods

### Ethics statements

This study was conducted in strict accordance with the recommendations provided in the Guide for the Care and Use of Laboratory Animals of the Ministry of Science and Technology of the People’s Republic of China. Animal experiments were approved by the Hubei Administrative Committee for Laboratory Animals (approval no. SYXK-2015-0084).

### Cells

HEK293T, A549, DF1, and MDCK (Madin-Darby canine kidney) cells were purchased from the American Type Culture Collection (Manassas, VA, USA), and MDCK MAVS knockout cells, maintained in RPMI 1640 (SH30809.01, HyClone) medium or Dulbecco’s modified Eagle’s medium (Gibco, NY, USA) or Ham’s/F-12 (SH30026.01, HyClone) medium supplemented with 10% heat-inactivated fetal bovine serum (522 PAN-Biotech, P30-3302), and incubated in 37°C humidified incubator with 5% CO_2_.

### Antibodies and reagents

Antibodies used in the study included the following: rabbit anti-YTHDC1 (GTX32976, GeneTex, USA); rabbit anti-YTHDF2 (24744-1-AP, proteintech); rabbit anti-METTL3 (15073-1-AP, proteintech); mouse anti-Flag-tag (F1804, SigmaAldrich, Saint Louis, MO, USA); mouse anti-HA-tag (M180-3, MBL, Japan); mouse anti-glyceraldehyde-3-phosphate dehydrogenase (GAPDH) (CB100127, California Bioscience, Coachella, CA, USA); rabbit anti-IAV NP, NS1, and NEP, (GTX125989, GTX125990, and GTX125953, GeneTex, USA); mouse anti-NP (produced in our laboratory); control rabbit IgG polyclonal (AC005, ABclonal Biotechnology, Cambridge, MA, USA); horseradish peroxidase-conjugated anti-mouse and anti-rabbit (BF03001 and BF03008, Beijing Biodragon Inmmunotechnologies, China); Alexa Fluor 594-conjugated goat anti-rabbit (GR200G-43C, Sungene Biotech); fluorescein isothiocyanate (FITC) goat anti-mouse (GM200G-02C, Sungene Biotech); and FITC-goat anti-rabbit (GR200G-02C, Sungene Biotech). 4′,6′-diamidino-2-phenylindole (1:1000) (no. C1002) was purchased from the Beyotime, China.

### IP-Mass spectrometry

Flag-tagged YTHDC1 or the empty vector was overexpressed in HEK293T cell lines for 24 h. Then, HEK293T cells were infected with 0.01 MOI PR8 virus for 24 h. Cells were washed three times with pre-cooled PBS and lysed in ice-cold radioimmunoprecipitation assay (RIPA) buffer (50 mM Tris pH 7.6, 150 mM NaCl, 2mM EDTA, 0.5 Nonidet P-40, 1 mM protease inhibitor (PMSF) and protease inhibitor cocktail). The cells were scraped off from the petri dish using a cell scraper and transferred to an ice-cold 1.5 mL EP tube. For fully lysed, the cells were slowly rotated at 4°C for 15–30 min and then centrifuged at 12,000×g for 15 min at 4°C. The cell lysates were transferred to a new Eppendorf tube and the cell debris was discarded. Flag M2 agarose beads (Sigma) or NS1 agarose beads were washed with RIPA buffer for three times and then resuspended by the same volume. Every 1 mL of cell lysates was added with 40 μL pre-mixed solution of anti-FLAG M2 or anti-NS1 agarose beads and then incubated with rotation for 6–8 h at 4°C, followed by centrifugation at 3,000×g at 4°C for 5 min to obtain the immunoprecipitates. After washing three times with 1 mL ice-cold RIPA buffer, the immunoprecipitates were resuspended in 40 μL 2×loading buffer and then boiled for 10min. A part of the sample was sent to the company for mass spectrometric detection and the remainder was stored at -80°C for further use. Analysis of the Mass Spectrometry data by bioinformatics. In this pipeline, results from search engines were pre-processed and re-scored using Percolator to improve the matching accuracy. The output was then filtered by FDR 1% at spectral level (PSM-level FDR < = 0.01) to obtain a significant identified spectrum and peptide list.

### Coimmunoprecipitation and western blotting

For RIP, A549 cells were infected with either the PR8 virus or its mutant at a multiplicity of infection (MOI) of 1 for 24 hours. Cell lysates were incubated with antibodies against either the YTHDC1, YTHDF2, or control IgG antibodies at 4°C overnight and precipitated with Dynabeads (Sc-2003, Santa Cruz Biotechnology, USA) at room temperature for 30 min. After three washes with RIP buffer, RNAs were purified and analyzed by RT-qPCR. For protein CoIP, HEK293T cells were cotransfected with indicated plasmids for 24 hours, or A549 cells were infected with the indicated virus at an MOI of 0.01; then, the cells were washed with cold phosphate-buffered saline (PBS) and lysed in cell lysis buffer for western blotting and immunoprecipitation (P0013, Beyotime) containing protease inhibitor cocktail (04693132001, Roche, Basel, Switzerland). Cell lysates were incubated with antibody-bead complexes at 4°C overnight. Protein-antibody-bead complexes were washed three times with immunoprecipitation lysis buffer as described above. The complexes were then mock-treated or treated with ribonuclease A/T (TaKaRa, 12091039; 1:1000) at 37°C for 1 hour, washed three times, and analyzed by western blotting.

### Immunofluorescence analysis

A549 cells were transfected with indicated plasmids with Lipofectamine 2000 (Invitrogen) according to the manufacturer’s protocol. Cells were washed three times with phosphate-buffered saline (PBS) and then fixed with 4% (wt/vol) paraformaldehyde for 15 min at room temperature. Cells were then washed three times with PBS and incubated with 0.1% Triton X-100 for 10 min. Next, 5% bovine serum albumin was used to block for 2 h. Cells were then incubated in Alexa Fluor FITC-conjugated goat anti-mouse (Flag and NS1) or 594-conjugated goat anti-rabbit (HA and YTHDC1) secondary antibodies for 2 h. Nuclei were stained with DAPI (00–4959–52, Invitrogen) for 10 min. All cells were washed with PBS 5 times after each step and were imaged by confocal microscopy (Carl Zeiss LSM 880 Confocal Microscope and ZEN 2.3 LITE software).

### Short hairpin (sh) RNA-mediated gene silencing

shRNAs specific to each gene used in the study described in previous study [26]. They were cloned into the pLKO.1-TRC vector kindly provided by Dr. Guan (Wuhan Institute of Virology, China). Stable knockdown cell lines were generated by lentiviral infection followed by puromycin selection. HEK293T cells were selected under puromycin at 5 μg/ml, while A549 cells were at 2.5 μg/ml.

### YTHDC1 rescue assay

shNC and shYTHDC1 A549 cells were transfected with empty vector or YTHDC1 for 24 h. Then cells washed with Ham’s/F-12 and infected with the indicated virus at an MOI of 0.01. The inoculums were removed after 1 hour of virus adsorption. Cells were then washed with Ham’s/F-12 and cultured in minimal essential media containing N-tosyl-L-phenylalanine chloromethyl ketone (TPCK) trypsin (0.25 μg/ml). Supernatants of infected cells were collected at 24 hpi and titrated on MDCK cells. Virus titers were determined by calculating log_10_TCID_50_/ml using the Reed and Muench method. Cell lysates were analyzed by western blotting.

### Cell viability assay

Cell viabilities were assessed using a cell counting kit-8 (CCK-8) (Cat NO. GK10001, GLPBIO, Montclair, CA, USA). Assays were performed according to the manufacturer’s instructions. Briefly, the shNC cells and the shYTHDC1 cells were seeded in 96-well plates and the cell viabilities were measured at 24 h. A total of 10 μL of CCK-8 reagents were added to each well of the plates, and the cells were incubated at 37°C for 1 h, then the absorbance at 450 nm was measured by a microplate reader.

### Growth kinetics of viruses

A549 cells were washed with Ham’s/F-12 and infected with the indicated virus at an MOI of 0.01. The inoculums were removed after 1 hour of virus adsorption. Cells were then washed with Ham’s/F-12 and cultured in minimal essential media containing N-tosyl-L-phenylalanine chloromethyl ketone (TPCK) trypsin (0.25 μg/ml). Supernatants of infected cells were collected at 12, 24, and 36 hpi and titrated on MDCK cells. Virus titers were determined by calculating log_10_TCID_50_/ml using the Reed and Muench method.

### RNA purification and RT-qPCR

According to the manufacturer’s instructions, total RNA was isolated from A549 cells with TRIzol Reagent (TaKaRa). The DNA present in total RNA was eliminated by DNase treatment (TaKaRa) by the user manual. RNA was transcribed into cDNA by reverse transcriptase (AMV XL, TaKaRa, Japan). RT-qPCR (ABI ViiA7, USA) was performed using SYBR Green 1 (Roche). GAPDH expression was detected in each sample and used for the normalization of gene expression between different samples. The PCR conditions were 2 min at 50°C, 10 min at 95°C, then 40 cycles of 15 s at 95°C, and 1 min at 60°C. The specificity of the assay was confirmed by melting curve analysis at the end of the amplification program (65°C to 95°C, 0.1°Cs-1). The primers used in this study were shown in S3 Table.

### Mini genome polymerase assay

Pol-I-NS-null plasmid construction. The whole NS sequence was cloned into a pol-I vector. And then mutated two stop codons at position 45 and 456. HEK293T cells grown in 12-well plates were cotransfected with reporter plasmid pol-I-NS-firefly (or pol-I-NS-null), virus polymerase, and NP plasmids (0.25 μg per well); plasmid pGL4.75 (0.02 μg per well; hRluc/CMV); and HA-YTHDC1 and HA-NS1-expressing plasmid (0.8 μg per well) or an empty vector for 24 hours. All RNAs were collected and tested for the related gene expression.

### RNA in vitro transcription, labeling, and pull-down assay

Wild type and NS_A530C_ mutant full-length of NS segment were amplified, which contained the T7 promoter at 5′. mRNA was synthesized by in vitro transcription with T7 RNA polymerase (M0251S, NEB, USA), and then, the DNA present in total RNA was eliminated by deoxyribonuclease (DNase) treatment (TaKaRa) following the user manual. mRNA was transcribed into cDNA by reverse transcriptase (AMV XL, TaKaRa, Japan), and then, mRNAs were labeled with biotin by using the Pierce RNA 3′ End Desthiobiotinylation Kit (20163, Thermo Fisher Scientific, USA). The labeled probes with biotin (TSINGKE Biological Technology, China) and labeled RNAs were incubated with A549 cell nuclear extracts; then, the RNA pull-down assay was performed using the Pierce Magnetic RNA Protein Pull-Down Kit (20164, Thermo Fisher Scientific, USA). The proteins were analyzed by western blotting.

### Viruses and reverse genetics

Recombinant viruses were generated in the genetic background of the PR8 virus using eight plasmid-based reverse genetic systems as described previously [44]. Eight segments of A/Puerto Rico/8/34 (H1N1) (PR8) were cloned into pHW2000 by our laboratory. The mutant NS gene at position 530 was generated by using a PCR-based site-directed mutagenesis. All constructs were confirmed by sequencing to ensure the absence of unwanted mutations. Recombinant viruses were propagated by a single passage in embryonic chicken eggs. Virus stocks of WT and recombinant viruses were sequenced to confirm no additional mutations.

### Pathogenicity study in mice

To determine MLD_50_ values, groups of five 6-week-old female BALB/c mice were lightly anesthetized with CO_2_ and inoculated intranasally with 10-fold serial dilutions of WT and NS_A530C_ in a volume of 50 μl. Mice were monitored daily for weight loss and mortality for 14 days. Mice that lost ≥20% of initial weight were humanely euthanized. MLD_50_ was calculated using the Reed and Muench method. To determine virus replication, groups of three BALB/c mice were intranasally inoculated with the indicated doses of WT and NS_A530C_ diluted in PBS and were euthanized on 3 and 5 dpi. Virus titers in lungs collected from infected mice were determined in chicken embryonated eggs.

### Statistical analysis

Virus titers in mouse mortality were statistically analyzed by Log-rank (Mantel Cox) test. Virus titers and RNA splicing were statistically analyzed by a two-way ANOVA test. The other data were statistically analyzed by one-way ANOVA test and student t-test.

## Supporting information

S1 Fig Identification proteins interact with NS1. (A) Schematic strategy for purification and identification of NS1 binding proteins via IP assay. Plasmid expressing Flag-tagged NS1 was transient transfection into HEK293T cells. HEK293T cells were infected or mock-infected with the PR8 virus. Cell lysates were performed for affinity purification by immunoprecipitation with protein A/G beads connected with Flag or NS1 antibody. The purified elutes were boiled in the SDS-PAGE loading buffer and then analyzed by MS. (B) Venn diagram showing the overlaps of differentially candidate NS1 binding proteins either in transfected or infected HEK293T cells. (C) A549 cells were transfected with indicated plasmids. Twenty-four hours after transfection, cells were fixed, permeabilized, and probed with anti-Flag/HA antibodies. FITC and Cy3 were used to visualize the indicated proteins. Diamidino-2-phenylindole shows the nuclei of cells. (D) The normalized fluorescence of YTHDC1 and NS1 along the white arrowheads shows overlapping peaks.
(TIF)

S2 Fig IAV infection promotes YTHDC1 expression. (A) A549 cells were infected with the PR8 virus at an MOI of 5 (top) or at an MOI of 0.01 (below). Cell lysates were collected at 3, 6, 9, 12, 24, and 36 hpi and subjected to western blotting analysis. (B) A549 cells were infected with the PR8 virus at MOI 0.01 for 24 h, mock-infected cells as a control, and YTHDC1 mRNA was determined by RT-qPCR which normalized to GAPDH mRNA. Data are presented as the average of three experiments and error bars indicate the standard error of the mean (SEM) (Student t-test; ns, not significant). (C) A549 cells were infected with the PR8 virus at an MOI of 0.01 for 24 h. Viral NP and host protein YTHDC1 were detected by the confocal assay. Diamidino-2-phenylindole shows the nuclei of cells. Data are presented as the average of 30 sights of the fluorescence which was calculated by ImageJ, and error bars indicate the standard error of the mean (SEM) (Student t-test, ***<0.001).
(TIF)

S3 Fig YTHDC1 did not change the NEP mRNA level and NS1 mRNA stability. (A) WT and YTHDC1 knockdown A549 cells were seeded on the 96-well plates, and cell viability was detected by Cell Counting Kit-8 assay at 24 hours. Data are presented as the average of eight experiments and error bars indicate the standard error of the mean (SEM) (Student t-test; ns, not significant). (B-F) The effect of YTHDC1 silencing on IAV RNA synthesis during infection. WT and YTHDC1 knockdown A549 cells were infected with the PR8 virus at MOI of 5. Samples were collected at 3, 6, and 9 hpi. The levels of NP (mRNA, cRNA, and vRNA), NS1, and NEP RNAs were determined by RT-qPCR. The viral RNA levels were normalized to the GAPDH mRNA level. Data are presented as the average of three experiments and error bars indicate the standard error of the mean (SEM) (two-way ANOVA test; ns, not significant; *, P<0.05; **, P<0.01; ***, P<0.001). (G) A549 cells were transfected with YTHDC1 and then infected with the PR8 virus at MOI of 5. Total RNA was collected at 6 hpi and subjected to RT-qPCR analysis. Data are presented as the average of three experiments and error bars indicate the standard error of the mean (SEM) (Student t-test; ns, not significant). (H) shNC and shYTHDC1 A549 cells were transfected with PHW2000-NS plasmid for 24 hours, and total RNA was extracted and subjected to RT-qPCR analysis. The NS RNA levels were normalized to the GAPDH mRNA level. Data are presented as the average of three experiments and error bars indicate the standard error of the mean (SEM) (Student ttest; ns, not significant).
(TIF)

S4 Fig NSA530C attenuated on MAVS knockout MDCK cells. MAVS knockout MDCK cells were infected with WT and NSA530C virus for 12, 24, and 36 h. The virus titer was determined by TCID50. Data are presented as the average of three experiments and error bars indicate the standard error of the mean (SEM) (two-way ANOVA test; ns, not significant; *, P<0.05; ***, P<0.001).
(TIF)

S5 Fig MLD_50_ of the WT and mutant viruses. (A-D) Weight loss of mortality of mice infected with the same doses of WT and mutated viruses. Six-week-old BALB/c mice were intranasally infected with 10-fold serially diluted (2.6x10^0^-2.6x10^5^ PFU) wild-type (WT) or mutants of PR8. Body weight (left) and survival (right) were monitored daily for two weeks (n = 5). (E) RNA was extracted from the lungs of the infected mouse at 5 dpi. NS1 was amplified and subjected to sanger sequencing. The area of 530 was shown in a black box.
(TIF)

S6 Fig Analysis of GGAC motif NS segment of IAV. The ratio of ISS in the NS segment (with GGAC number/without GGAC number/total of numbers analyzed) in each analyzed subtype of IAVs isolated from human and avian species. Both human H5 (1821/0/1821), H7 (1035/0/1035), and H9 (15/0/15), and avian H5 (3930/28/3958), H7 (1438/132/1570), and H9 (2617/8/2625) viruses.
(TIF)

S1 Table Proteins pulled down by NS1 and Flag-NS1. (DOCX)

S2 Table Analysis of GGAC motif in NS segment of H5, H7 and H9 IAVs. (DOCX)

S3 Table Primers and probes. (DOCX)
