# Supplementary figures and images for: N6-methyladenosine reader protein YTHDC1 regulates influenza A virus NS segment splicing and replication
Source: PLoS Pathog. 2023 Apr 13;19(4):e1011305. doi: 10.1371/journal.ppat.1011305 (PMC10146569; doi:10.1371/journal.ppat.1011305)

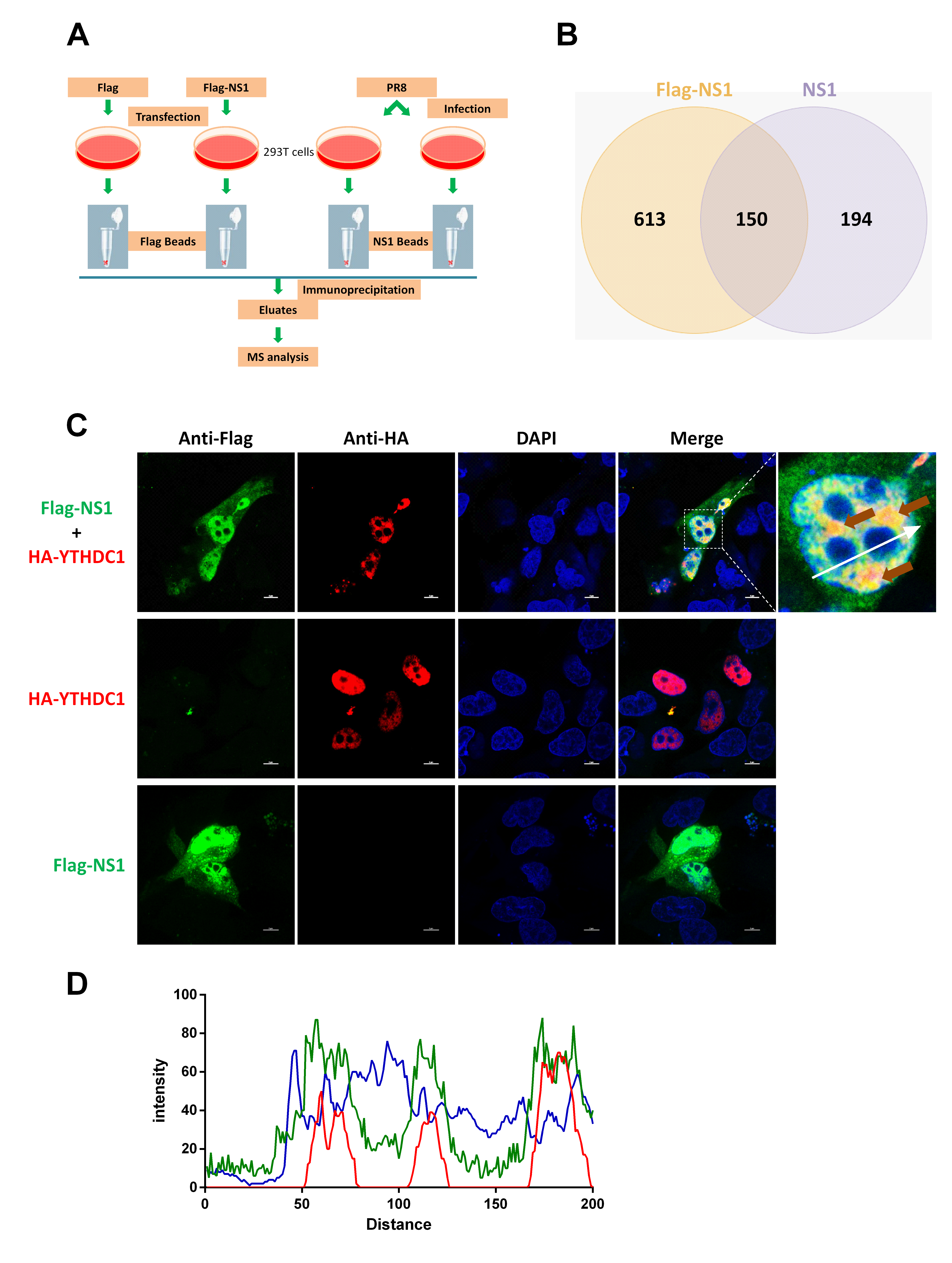

Supplement: S1 Fig — (A) Schematic strategy for purification and identification of NS1 binding proteins via IP assay. Plasmid expressing Flag-tagged NS1 was transient transfection into HEK293T cells. HEK293T cells were infected or mock-infected with the PR8 virus. Cell lysates were performed for affinity purification by immunoprecipitation with protein A/G beads connected with Flag or NS1 antibody. The purified elutes were boiled in the SDS-PAGE loading buffer and then analyzed by MS. (B) Venn diagram showing the overlaps of differentially candidate NS1 binding proteins either in transfected or infected HEK293T cells. (C) A549 cells were transfected with indicated plasmids. Twenty-four hours after transfection, cells were fixed, permeabilized, and probed with anti-Flag/HA antibodies. FITC and Cy3 were used to visualize the indicated proteins. Diamidino-2-phenylindole shows the nuclei of cells. (D) The normalized fluorescence of YTHDC1 and NS1 along the white arrowheads shows overlapping peaks. (TIF) [file ppat.1011305.s001.tif]

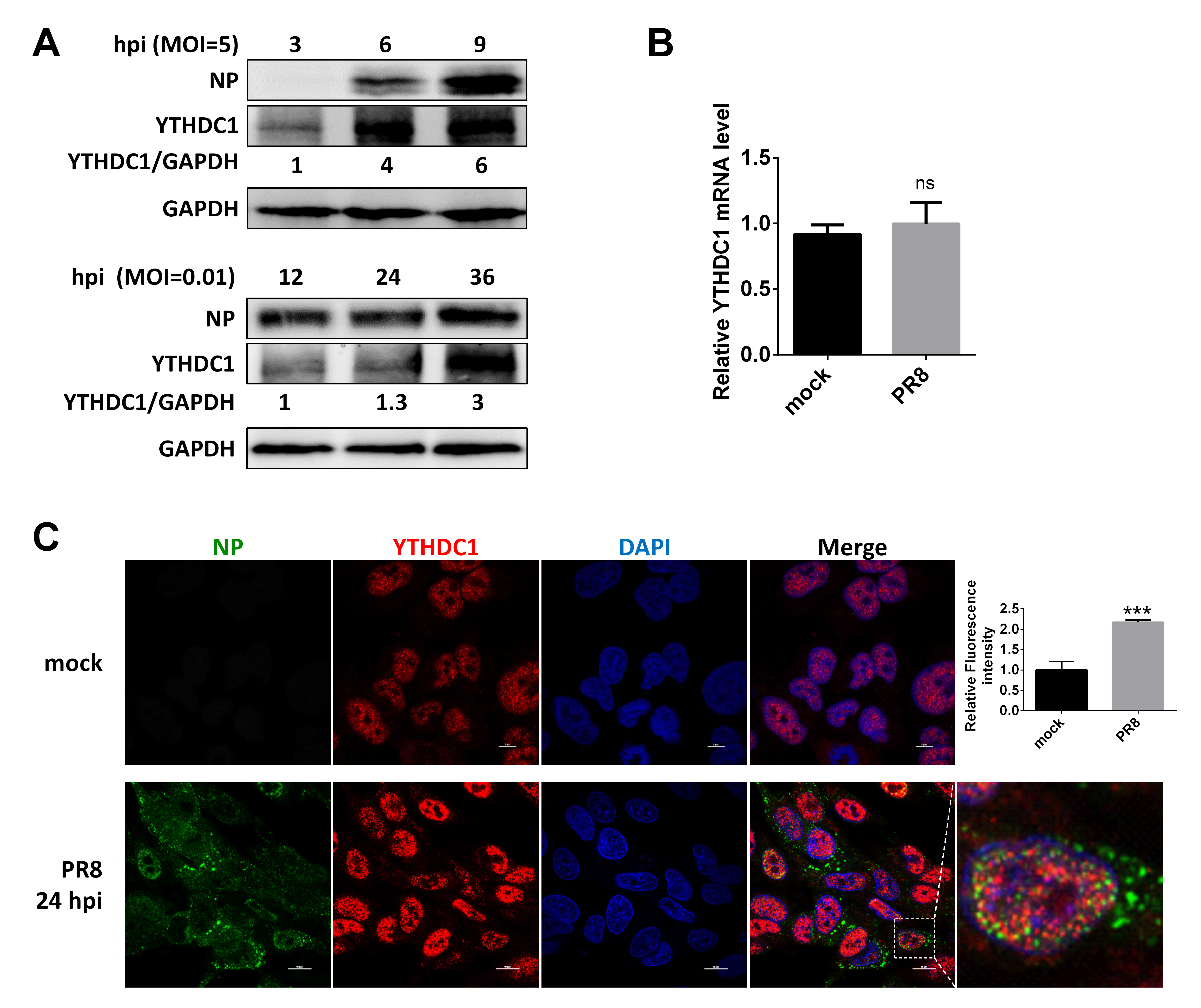

Supplement: S2 Fig — (A) A549 cells were infected with the PR8 virus at an MOI of 5 (top) or at an MOI of 0.01 (below). Cell lysates were collected at 3, 6, 9, 12, 24, and 36 hpi and subjected to western blotting analysis. (B) A549 cells were infected with the PR8 virus at MOI 0.01 for 24 h, mock-infected cells as a control, and YTHDC1 mRNA was determined by RT-qPCR which normalized to GAPDH mRNA. Data are presented as the average of three experiments and error bars indicate the standard error of the mean (SEM) (Student t-test; ns, not significant). (C) A549 cells were infected with the PR8 virus at an MOI of 0.01 for 24 h. Viral NP and host protein YTHDC1 were detected by the confocal assay. Diamidino-2-phenylindole shows the nuclei of cells. Data are presented as the average of 30 sights of the fluorescence which was calculated by ImageJ, and error bars indicate the standard error of the mean (SEM) (Student t-test, ***<0.001). (TIF) [file ppat.1011305.s002.tif]

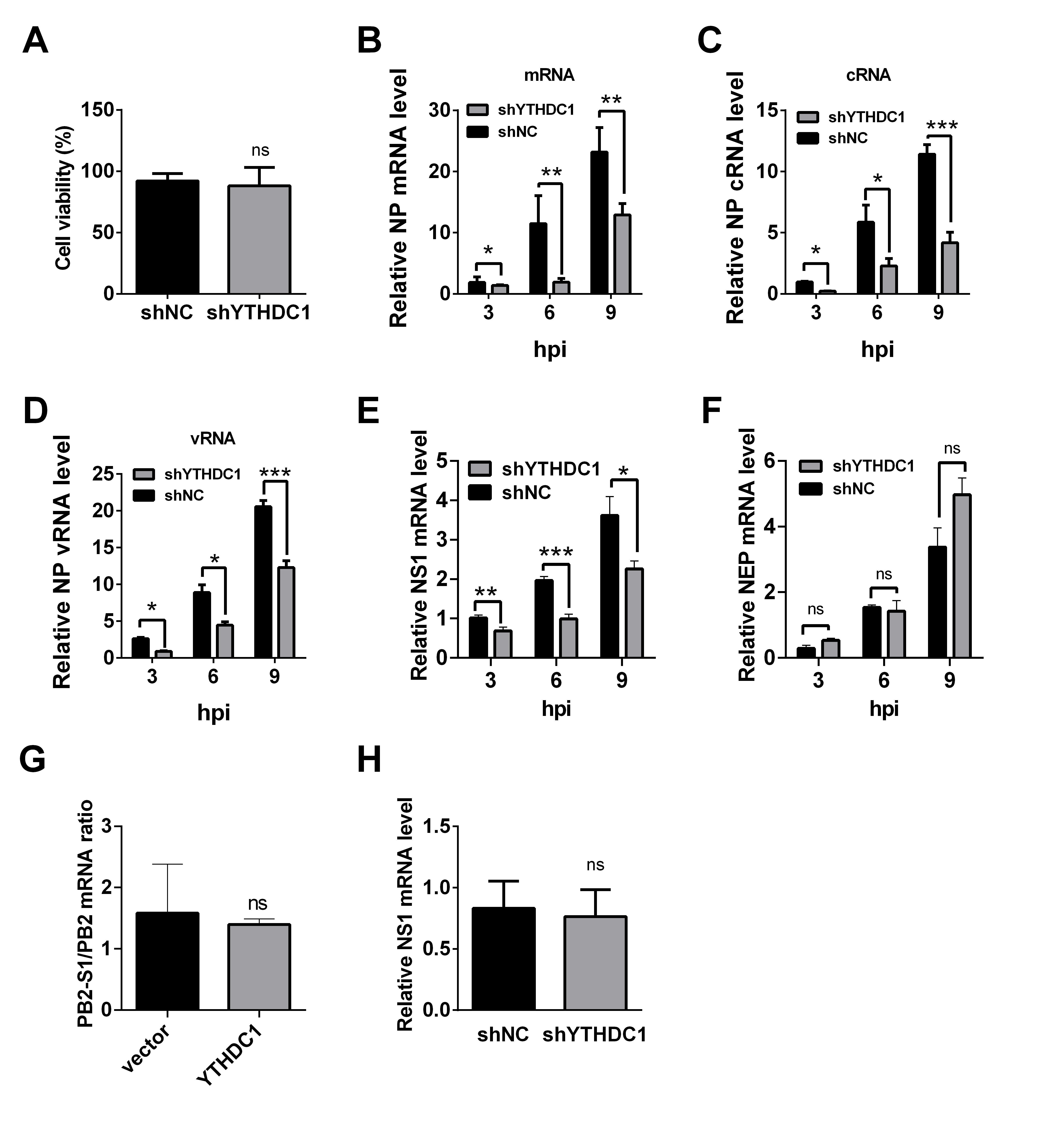

Supplement: S3 Fig — (A) WT and YTHDC1 knockdown A549 cells were seeded on the 96-well plates, and cell viability was detected by Cell Counting Kit-8 assay at 24 hours. Data are presented as the average of eight experiments and error bars indicate the standard error of the mean (SEM) (Student t-test; ns, not significant). (B-F) The effect of YTHDC1 silencing on IAV RNA synthesis during infection. WT and YTHDC1 knockdown A549 cells were infected with the PR8 virus at MOI of 5. Samples were collected at 3, 6, and 9 hpi. The levels of NP (mRNA, cRNA, and vRNA), NS1, and NEP RNAs were determined by RT-qPCR. The viral RNA levels were normalized to the GAPDH mRNA level. Data are presented as the average of three experiments and error bars indicate the standard error of the mean (SEM) (two-way ANOVA test; ns, not significant; *, P<0.05; **, P<0.01; ***, P<0.001). (G) A549 cells were transfected with YTHDC1 and then infected with the PR8 virus at MOI of 5. Total RNA was collected at 6 hpi and subjected to RT-qPCR analysis. Data are presented as the average of three experiments and error bars indicate the standard error of the mean (SEM) (Student t-test; ns, not significant). (H) shNC and shYTHDC1 A549 cells were transfected with PHW2000-NS plasmid for 24 hours, and total RNA was extracted and subjected to RT-qPCR analysis. The NS RNA levels were normalized to the GAPDH mRNA level. Data are presented as the average of three experiments and error bars indicate the standard error of the mean (SEM) (Student ttest; ns, not significant). (TIF) [file ppat.1011305.s003.tif]

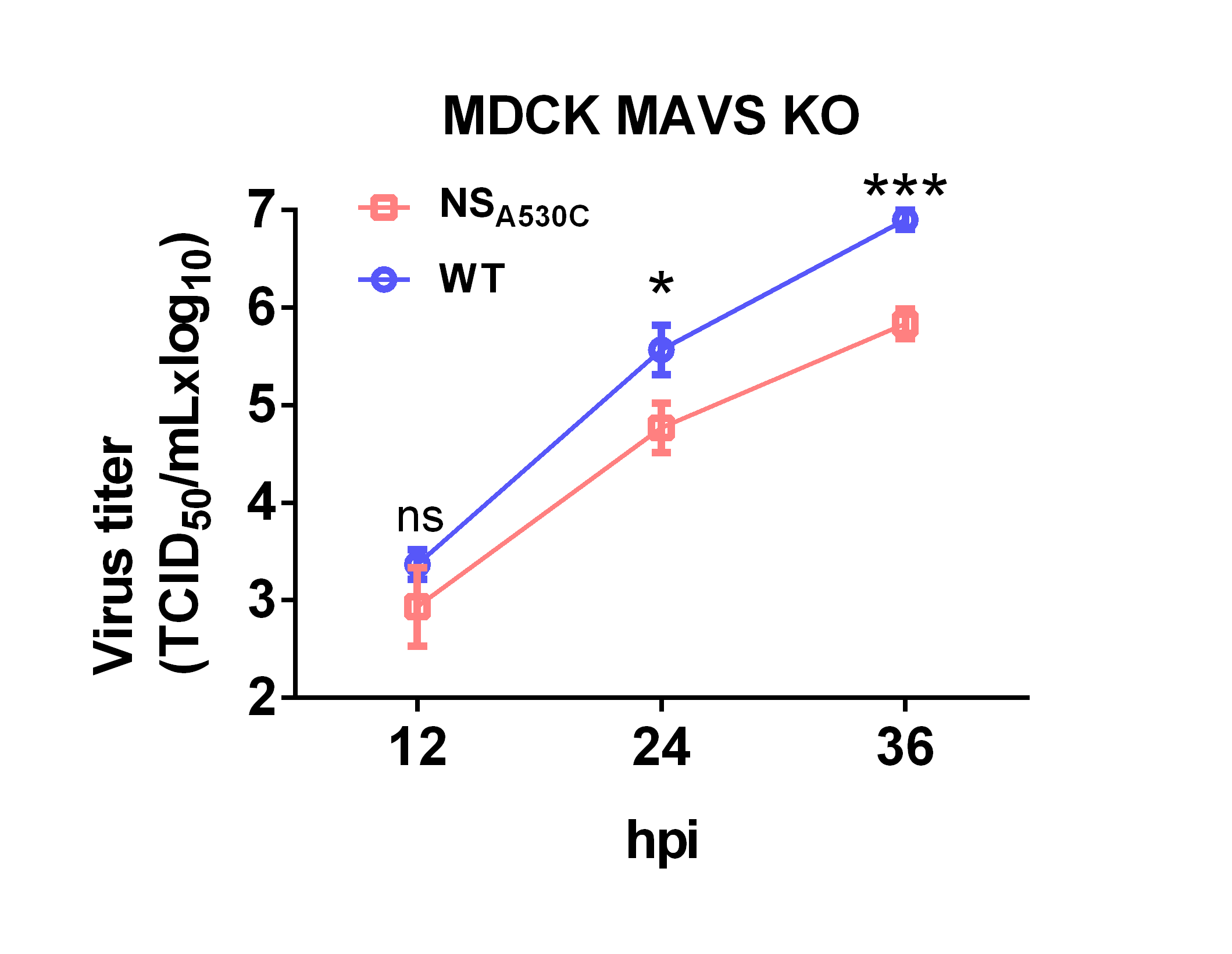

Supplement: S4 Fig — MAVS knockout MDCK cells were infected with WT and NSA530C virus for 12, 24, and 36 h. The virus titer was determined by TCID50. Data are presented as the average of three experiments and error bars indicate the standard error of the mean (SEM) (two-way ANOVA test; ns, not significant; *, P<0.05; ***, P<0.001). (TIF) [file ppat.1011305.s004.tif]

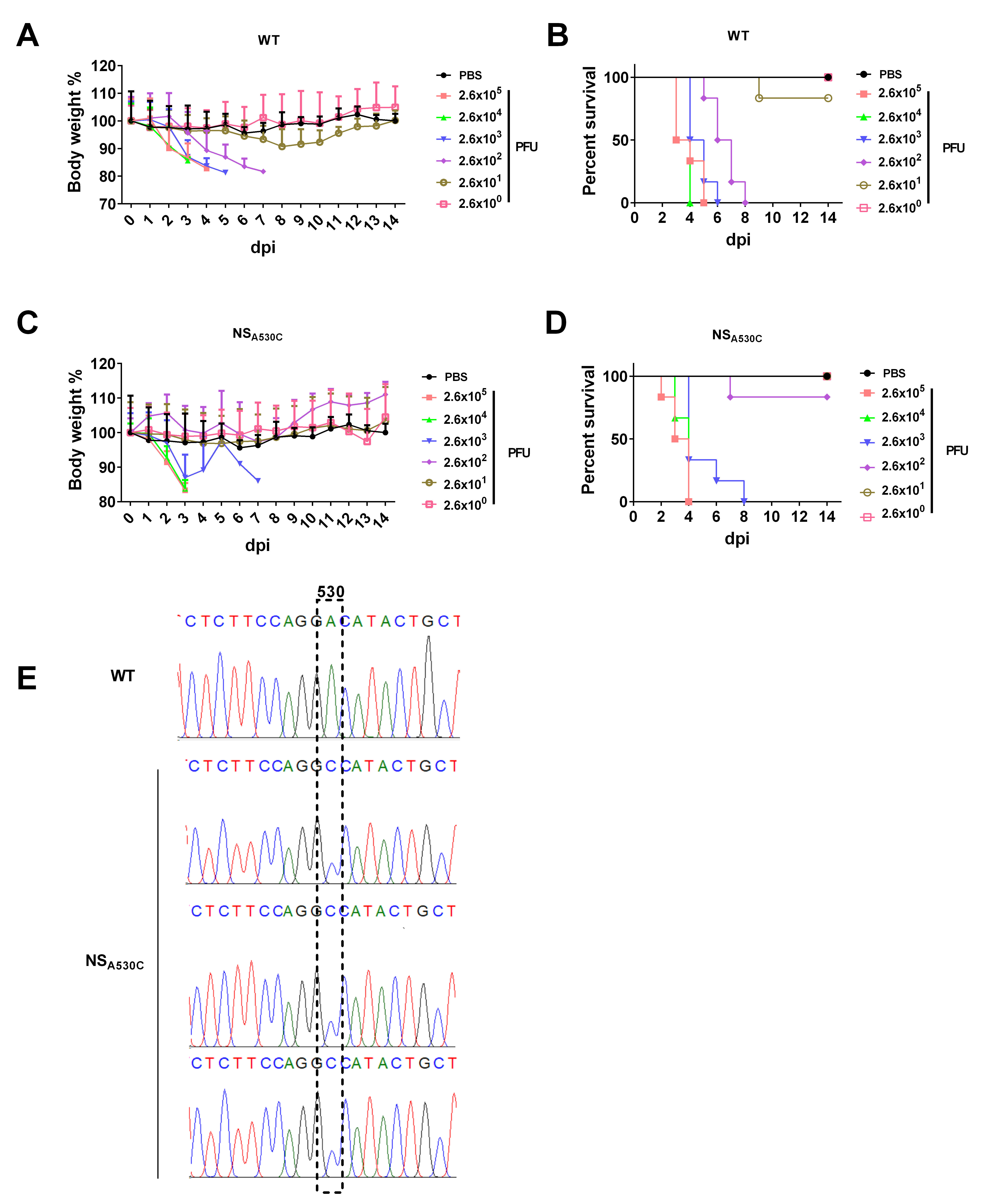

Supplement: S5 Fig — (A-D) Weight loss of mortality of mice infected with the same doses of WT and mutated viruses. Six-week-old BALB/c mice were intranasally infected with 10-fold serially diluted (2.6x100-2.6x10^5^ PFU) wild-type (WT) or mutants of PR8. Body weight (left) and survival (right) were monitored daily for two weeks (n = 5). (E) RNA was extracted from the lungs of the infected mouse at 5 dpi. NS1 was amplified and subjected to sanger sequencing. The area of 530 was shown in a black box. (TIF) [file ppat.1011305.s005.tif]

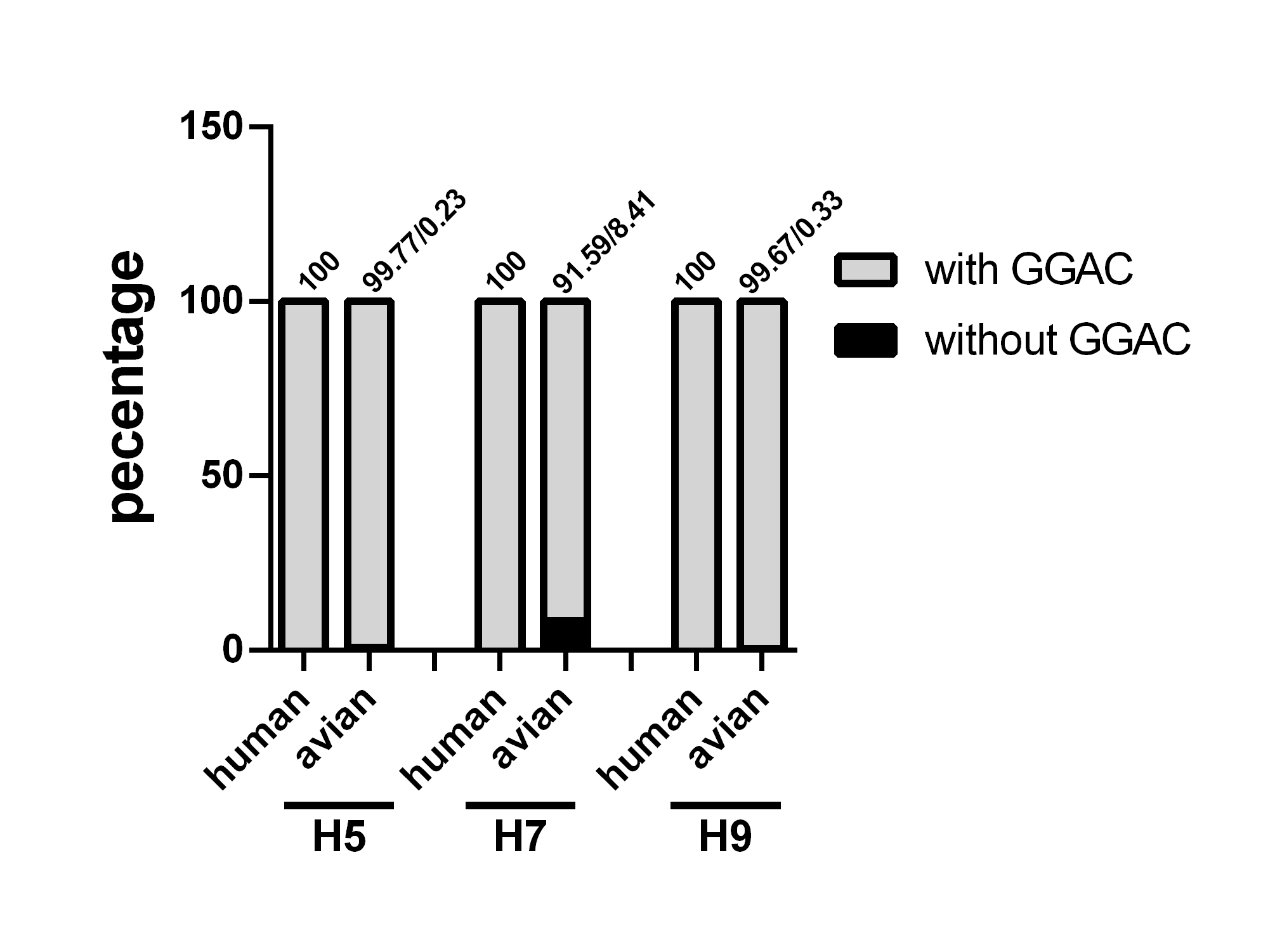

Supplement: S6 Fig — The ratio of ISS in the NS segment (with GGAC number/without GGAC number/total of numbers analyzed) in each analyzed subtype of IAVs isolated from human and avian species. Both human H5 (1821/0/1821), H7 (1035/0/1035), and H9 (15/0/15), and avian H5 (3930/28/3958), H7 (1438/132/1570), and H9 (2617/8/2625) viruses. (TIF) [file ppat.1011305.s006.tif]
