# Supplementary material for: N6-methyladenosine reader protein YTHDC1 regulates influenza A virus NS segment splicing and replication
Source: PLoS Pathog. 2023 Apr 13;19(4):e1011305. doi: 10.1371/journal.ppat.1011305 (PMC10146569; doi:10.1371/journal.ppat.1011305)
Supplement: S1 Table — (DOCX) [file ppat.1011305.s007.docx]

**S1 Table. Proteins pulled down by NS1 and Flag-NS1.**

| Protein | Description |
| --- | --- |
| MYH14 | Myosin-14 OS=Homo sapiens OX=9606 GN=MYH14 PE=1 SV=2 |
| MY18A | Unconventional myosin-XVIIIa OS=Homo sapiens OX=9606 GN=MYO18A PE=1 SV=3 |
| MYO5A | Unconventional myosin-Va OS=Homo sapiens OX=9606 GN=MYO5A PE=1 SV=2 |
| SI1L1 | Signal-induced proliferation-associated 1-like protein 1 OS=Homo sapiens OX=9606 GN=SIPA1L1 PE=1 SV=4 |
| FLII | Protein flightless-1 homolog OS=Homo sapiens OX=9606 GN=FLII PE=1 SV=2 |
| SI1L3 | Signal-induced proliferation-associated 1-like protein 3 OS=Homo sapiens OX=9606 GN=SIPA1L3 PE=1 SV=3 |
| NEB2 | Neurabin-2 OS=Homo sapiens OX=9606 GN=PPP1R9B PE=1 SV=3 |
| MYO6 | Unconventional myosin-VI OS=Homo sapiens OX=9606 GN=MYO6 PE=1 SV=4 |
| SRC8 | Src substrate cortactin OS=Homo sapiens OX=9606 GN=CTTN PE=1 SV=2 |
| LMO7 | LIM domain only protein 7 OS=Homo sapiens OX=9606 GN=LMO7 PE=1 SV=3 |
| CING | Cingulin OS=Homo sapiens OX=9606 GN=CGN PE=1 SV=2 |
| MYO5C | Unconventional myosin-Vc OS=Homo sapiens OX=9606 GN=MYO5C PE=1 SV=2 |
| LRRF2 | Leucine-rich repeat flightless-interacting protein 2 OS=Homo sapiens OX=9606 GN=LRRFIP2 PE=1 SV=1 |
| TMOD1 | Tropomodulin-1 OS=Homo sapiens OX=9606 GN=TMOD1 PE=1 SV=1 |
| MYL6B | Myosin light chain 6B OS=Homo sapiens OX=9606 GN=MYL6B PE=1 SV=1 |
| PCM1 | Pericentriolar material 1 protein OS=Homo sapiens OX=9606 GN=PCM1 PE=1 SV=5 |
| ARP3 | Actin-related protein 3 OS=Homo sapiens OX=9606 GN=ACTR3 PE=1 SV=3 |
| ARPC2 | Actin-related protein 2/3 complex subunit 2 OS=Homo sapiens OX=9606 GN=ARPC2 PE=1 SV=1 |
| DSG2 | Desmoglein-2 OS=Homo sapiens OX=9606 GN=DSG2 PE=1 SV=2 |
| MYPT2 | Protein phosphatase 1 regulatory subunit 12B OS=Homo sapiens OX=9606 GN=PPP1R12B PE=1 SV=2 |
| SPTN2 | Spectrin beta chain, non-erythrocytic 2 OS=Homo sapiens OX=9606 GN=SPTBN2 PE=1 SV=3 |
| CYTSB | Cytospin-B OS=Homo sapiens OX=9606 GN=SPECC1 PE=1 SV=1 |
| CALD1 | Caldesmon OS=Homo sapiens OX=9606 GN=CALD1 PE=1 SV=3 |
| CAZA2 | F-actin-capping protein subunit alpha-2 OS=Homo sapiens OX=9606 GN=CAPZA2 PE=1 SV=3 |
| ARP2 | Actin-related protein 2 OS=Homo sapiens OX=9606 GN=ACTR2 PE=1 SV=1 |
| CP131 | Centrosomal protein of 131 kDa OS=Homo sapiens OX=9606 GN=CEP131 PE=1 SV=3 |
| ARC1A | Actin-related protein 2/3 complex subunit 1A OS=Homo sapiens OX=9606 GN=ARPC1A PE=2 SV=2 |
| EFHD2 | EF-hand domain-containing protein D2 OS=Homo sapiens OX=9606 GN=EFHD2 PE=1 SV=1 |
| ABLM1 | Actin-binding LIM protein 1 OS=Homo sapiens OX=9606 GN=ABLIM1 PE=1 SV=3 |
| RRAGC | Ras-related GTP-binding protein C OS=Homo sapiens OX=9606 GN=RRAGC PE=1 SV=1 |
| YTDC1 | YTH domain-containing protein 1 OS=Homo sapiens OX=9606 GN=YTHDC1 PE=1 SV=3 |
| DBNL | Drebrin-like protein OS=Homo sapiens OX=9606 GN=DBNL PE=1 SV=1 |
| ITPI2 | Protein ITPRID2 OS=Homo sapiens OX=9606 GN=ITPRID2 PE=1 SV=3 |
| TOP2A | DNA topoisomerase 2-alpha OS=Homo sapiens OX=9606 GN=TOP2A PE=1 SV=3 |
| SSRP1 | FACT complex subunit SSRP1 OS=Homo sapiens OX=9606 GN=SSRP1 PE=1 SV=1 |
| TMOD2 | Tropomodulin-2 OS=Homo sapiens OX=9606 GN=TMOD2 PE=1 SV=1 |
| ITPR2 | Inositol 1,4,5-trisphosphate receptor type 2 OS=Homo sapiens OX=9606 GN=ITPR2 PE=1 SV=2 |
| RHG21 | Rho GTPase-activating protein 21 OS=Homo sapiens OX=9606 GN=ARHGAP21 PE=1 SV=2 |
| PHAG1 | Phosphoprotein associated with glycosphingolipid-enriched microdomains 1 OS=Homo sapiens OX=9606 GN=PAG1 PE=1 SV=2 |
| CADH2 | Cadherin-2 OS=Homo sapiens OX=9606 GN=CDH2 PE=1 SV=4 |
| TCOF | Treacle protein OS=Homo sapiens OX=9606 GN=TCOF1 PE=1 SV=3 |
| CXA1 | Gap junction alpha-1 protein OS=Homo sapiens OX=9606 GN=GJA1 PE=1 SV=2 |
| MYH11 | Myosin-11 OS=Homo sapiens OX=9606 GN=MYH11 PE=1 SV=3 |
| LUZP1 | Leucine zipper protein 1 OS=Homo sapiens OX=9606 GN=LUZP1 PE=1 SV=2 |
| UN45A | Protein unc-45 homolog A OS=Homo sapiens OX=9606 GN=UNC45A PE=1 SV=1 |
| EFHD1 | EF-hand domain-containing protein D1 OS=Homo sapiens OX=9606 GN=EFHD1 PE=1 SV=1 |
| ACTBL | Beta-actin-like protein 2 OS=Homo sapiens OX=9606 GN=ACTBL2 PE=1 SV=2 |
| MIB1 | E3 ubiquitin-protein ligase MIB1 OS=Homo sapiens OX=9606 GN=MIB1 PE=1 SV=1 |
| TEAD1 | Transcriptional enhancer factor TEF-1 OS=Homo sapiens OX=9606 GN=TEAD1 PE=1 SV=2 |
| GNAQ | Guanine nucleotide-binding protein G(q) subunit alpha OS=Homo sapiens OX=9606 GN=GNAQ PE=1 SV=4 |
| ITPR3 | Inositol 1,4,5-trisphosphate receptor type 3 OS=Homo sapiens OX=9606 GN=ITPR3 PE=1 SV=2 |
| GNAI1 | Guanine nucleotide-binding protein G(i) subunit alpha-1 OS=Homo sapiens OX=9606 GN=GNAI1 PE=1 SV=2 |
| MICA3 | [F-actin]-monooxygenase MICAL3 OS=Homo sapiens OX=9606 GN=MICAL3 PE=1 SV=2 |
| FBRL | rRNA 2'-O-methyltransferase fibrillarin OS=Homo sapiens OX=9606 GN=FBL PE=1 SV=2 |
| CRAD | Cancer-related regulator of actin dynamics OS=Homo sapiens OX=9606 GN=CRAD PE=1 SV=3 |
| PKP4 | Plakophilin-4 OS=Homo sapiens OX=9606 GN=PKP4 PE=1 SV=2 |
| P85B | Phosphatidylinositol 3-kinase regulatory subunit beta OS=Homo sapiens OX=9606 GN=PIK3R2 PE=1 SV=2 |
| PLEC | Plectin OS=Homo sapiens OX=9606 GN=PLEC PE=1 SV=3 |
| GNA11 | Guanine nucleotide-binding protein subunit alpha-11 OS=Homo sapiens OX=9606 GN=GNA11 PE=1 SV=2 |
| STOM | Erythrocyte band 7 integral membrane protein OS=Homo sapiens OX=9606 GN=STOM PE=1 SV=3 |
| WDR1 | WD repeat-containing protein 1 OS=Homo sapiens OX=9606 GN=WDR1 PE=1 SV=4 |
| ARC1B | Actin-related protein 2/3 complex subunit 1B OS=Homo sapiens OX=9606 GN=ARPC1B PE=1 SV=3 |
| CALM1 | Calmodulin-1 OS=Homo sapiens OX=9606 GN=CALM1 PE=1 SV=1 |
| MYO5B | Unconventional myosin-Vb OS=Homo sapiens OX=9606 GN=MYO5B PE=1 SV=3 |
| RFA3 | Replication protein A 14 kDa subunit OS=Homo sapiens OX=9606 GN=RPA3 PE=1 SV=1 |
| BRX1 | Ribosome biogenesis protein BRX1 homolog OS=Homo sapiens OX=9606 GN=BRIX1 PE=1 SV=2 |
| EFR3A | Protein EFR3 homolog A OS=Homo sapiens OX=9606 GN=EFR3A PE=1 SV=2 |
| LZTS3 | Leucine zipper putative tumor suppressor 3 OS=Homo sapiens OX=9606 GN=LZTS3 PE=2 SV=1 |
| NEXN | Nexilin OS=Homo sapiens OX=9606 GN=NEXN PE=1 SV=1 |
| RFC1 | Replication factor C subunit 1 OS=Homo sapiens OX=9606 GN=RFC1 PE=1 SV=4 |
| LACTB | Serine beta-lactamase-like protein LACTB, mitochondrial OS=Homo sapiens OX=9606 GN=LACTB PE=1 SV=2 |
| TPRN | Taperin OS=Homo sapiens OX=9606 GN=TPRN PE=1 SV=2 |
| CTNA1 | Catenin alpha-1 OS=Homo sapiens OX=9606 GN=CTNNA1 PE=1 SV=1 |
| DDX18 | ATP-dependent RNA helicase DDX18 OS=Homo sapiens OX=9606 GN=DDX18 PE=1 SV=2 |
| BAF | Barrier-to-autointegration factor OS=Homo sapiens OX=9606 GN=BANF1 PE=1 SV=1 |
| RAB1B | Ras-related protein Rab-1B OS=Homo sapiens OX=9606 GN=RAB1B PE=1 SV=1 |
| SP16H | FACT complex subunit SPT16 OS=Homo sapiens OX=9606 GN=SUPT16H PE=1 SV=1 |
| DAPK1 | Death-associated protein kinase 1 OS=Homo sapiens OX=9606 GN=DAPK1 PE=1 SV=6 |
| LIMC1 | LIM and calponin homology domains-containing protein 1 OS=Homo sapiens OX=9606 GN=LIMCH1 PE=1 SV=4 |
| GPC6 | Glypican-6 OS=Homo sapiens OX=9606 GN=GPC6 PE=1 SV=1 |
| RALY | RNA-binding protein Raly OS=Homo sapiens OX=9606 GN=RALY PE=1 SV=1 |
| RBM28 | RNA-binding protein 28 OS=Homo sapiens OX=9606 GN=RBM28 PE=1 SV=3 |
| RRP44 | Exosome complex exonuclease RRP44 OS=Homo sapiens OX=9606 GN=DIS3 PE=1 SV=2 |
| NCKP1 | Nck-associated protein 1 OS=Homo sapiens OX=9606 GN=NCKAP1 PE=1 SV=1 |
| NEB1 | Neurabin-1 OS=Homo sapiens OX=9606 GN=PPP1R9A PE=1 SV=2 |
| SH3L2 | SH3 domain-binding glutamic acid-rich-like protein 2 OS=Homo sapiens OX=9606 GN=SH3BGRL2 PE=1 SV=2 |
| GAPR1 | Golgi-associated plant pathogenesis-related protein 1 OS=Homo sapiens OX=9606 GN=GLIPR2 PE=1 SV=3 |
| ZDHC5 | Palmitoyltransferase ZDHHC5 OS=Homo sapiens OX=9606 GN=ZDHHC5 PE=1 SV=2 |
| ARP5L | Actin-related protein 2/3 complex subunit 5-like protein OS=Homo sapiens OX=9606 GN=ARPC5L PE=1 SV=1 |
| ASM3B | Acid sphingomyelinase-like phosphodiesterase 3b OS=Homo sapiens OX=9606 GN=SMPDL3B PE=1 SV=2 |
| DNJB3 | DnaJ homolog subfamily B member 3 OS=Homo sapiens OX=9606 GN=DNAJB3 PE=1 SV=1 |
| LTOR1 | Ragulator complex protein LAMTOR1 OS=Homo sapiens OX=9606 GN=LAMTOR1 PE=1 SV=2 |
| GNAZ | Guanine nucleotide-binding protein G(z) subunit alpha OS=Homo sapiens OX=9606 GN=GNAZ PE=1 SV=3 |
| EFCB7 | EF-hand calcium-binding domain-containing protein 7 OS=Homo sapiens OX=9606 GN=EFCAB7 PE=1 SV=1 |
| DIM1 | Probable dimethyladenosine transferase OS=Homo sapiens OX=9606 GN=DIMT1 PE=1 SV=1 |
| LTOR3 | Ragulator complex protein LAMTOR3 OS=Homo sapiens OX=9606 GN=LAMTOR3 PE=1 SV=1 |
| ANLN | Anillin OS=Homo sapiens OX=9606 GN=ANLN PE=1 SV=2 |
| KC1G1 | Casein kinase I isoform gamma-1 OS=Homo sapiens OX=9606 GN=CSNK1G1 PE=1 SV=1 |
| PKP2 | Plakophilin-2 OS=Homo sapiens OX=9606 GN=PKP2 PE=1 SV=2 |
| SSH1 | Protein phosphatase Slingshot homolog 1 OS=Homo sapiens OX=9606 GN=SSH1 PE=1 SV=2 |
| MOXD1 | DBH-like monooxygenase protein 1 OS=Homo sapiens OX=9606 GN=MOXD1 PE=1 SV=1 |
| AIMP2 | Aminoacyl tRNA synthase complex-interacting multifunctional protein 2 OS=Homo sapiens OX=9606 GN=AIMP2 PE=1 SV=2 |
| GBG5 | Guanine nucleotide-binding protein G(I)/G(S)/G(O) subunit gamma-5 OS=Homo sapiens OX=9606 GN=GNG5 PE=1 SV=3 |
| CA2D1 | Voltage-dependent calcium channel subunit alpha-2/delta-1 OS=Homo sapiens OX=9606 GN=CACNA2D1 PE=1 SV=3 |
| GPC3 | Glypican-3 OS=Homo sapiens OX=9606 GN=GPC3 PE=1 SV=1 |
| SRP09 | Signal recognition particle 9 kDa protein OS=Homo sapiens OX=9606 GN=SRP9 PE=1 SV=2 |
| CXG1 | Gap junction gamma-1 protein OS=Homo sapiens OX=9606 GN=GJC1 PE=1 SV=2 |
| SDC2 | Syndecan-2 OS=Homo sapiens OX=9606 GN=SDC2 PE=1 SV=2 |
| MACF1 | Microtubule-actin cross-linking factor 1, isoforms 1/2/3/5 OS=Homo sapiens OX=9606 GN=MACF1 PE=1 SV=4 |
| TRM7 | Putative tRNA (cytidine(32)/guanosine(34)-2'-O)-methyltransferase OS=Homo sapiens OX=9606 GN=FTSJ1 PE=1 SV=2 |
| RCC2 | Protein RCC2 OS=Homo sapiens OX=9606 GN=RCC2 PE=1 SV=2 |
| MK67I | MKI67 FHA domain-interacting nucleolar phosphoprotein OS=Homo sapiens OX=9606 GN=NIFK PE=1 SV=1 |
| MFGM | Lactadherin OS=Homo sapiens OX=9606 GN=MFGE8 PE=1 SV=3 |
| PP1G | Serine/threonine-protein phosphatase PP1-gamma catalytic subunit OS=Homo sapiens OX=9606 GN=PPP1CC PE=1 SV=1 |
| CTNA2 | Catenin alpha-2 OS=Homo sapiens OX=9606 GN=CTNNA2 PE=1 SV=5 |
| ODB2 | Lipoamide acyltransferase component of branched-chain alpha-keto acid dehydrogenase complex, mitochondrial OS=Homo sapiens OX=9606 GN=DBT PE=1 SV=3 |
| PRIO | Major prion protein OS=Homo sapiens OX=9606 GN=PRNP PE=1 SV=1 |
| HNRL2 | Heterogeneous nuclear ribonucleoprotein U-like protein 2 OS=Homo sapiens OX=9606 GN=HNRNPUL2 PE=1 SV=1 |
| ITPR1 | Inositol 1,4,5-trisphosphate receptor type 1 OS=Homo sapiens OX=9606 GN=ITPR1 PE=1 SV=3 |
| COR2A | Coronin-2A OS=Homo sapiens OX=9606 GN=CORO2A PE=1 SV=2 |
| VAS1 | V-type proton ATPase subunit S1 OS=Homo sapiens OX=9606 GN=ATP6AP1 PE=1 SV=2 |
| P85A | Phosphatidylinositol 3-kinase regulatory subunit alpha OS=Homo sapiens OX=9606 GN=PIK3R1 PE=1 SV=2 |
| E41L5 | Band 4.1-like protein 5 OS=Homo sapiens OX=9606 GN=EPB41L5 PE=1 SV=3 |
| CUL2 | Cullin-2 OS=Homo sapiens OX=9606 GN=CUL2 PE=1 SV=2 |
| RL39 | 60S ribosomal protein L39 OS=Homo sapiens OX=9606 GN=RPL39 PE=1 SV=2 |
| NOC3L | Nucleolar complex protein 3 homolog OS=Homo sapiens OX=9606 GN=NOC3L PE=1 SV=1 |
| TPM2 | Tropomyosin beta chain OS=Homo sapiens OX=9606 GN=TPM2 PE=1 SV=1 |
| SRS11 | Serine/arginine-rich splicing factor 11 OS=Homo sapiens OX=9606 GN=SRSF11 PE=1 SV=1 |
| RBM5 | RNA-binding protein 5 OS=Homo sapiens OX=9606 GN=RBM5 PE=1 SV=2 |
| H2AY | Core histone macro-H2A.1 OS=Homo sapiens OX=9606 GN=H2AFY PE=1 SV=4 |
| DAPK3 | Death-associated protein kinase 3 OS=Homo sapiens OX=9606 GN=DAPK3 PE=1 SV=1 |
| REV1 | DNA repair protein REV1 OS=Homo sapiens OX=9606 GN=REV1 PE=1 SV=1 |
| GBB4 | Guanine nucleotide-binding protein subunit beta-4 OS=Homo sapiens OX=9606 GN=GNB4 PE=1 SV=3 |
| LRC27 | Leucine-rich repeat-containing protein 27 OS=Homo sapiens OX=9606 GN=LRRC27 PE=2 SV=2 |
| MYO1E | Unconventional myosin-Ie OS=Homo sapiens OX=9606 GN=MYO1E PE=1 SV=2 |
| SMCA1 | Probable global transcription activator SNF2L1 OS=Homo sapiens OX=9606 GN=SMARCA1 PE=1 SV=2 |
| GELS | Gelsolin OS=Homo sapiens OX=9606 GN=GSN PE=1 SV=1 |
| PSIP1 | PC4 and SFRS1-interacting protein OS=Homo sapiens OX=9606 GN=PSIP1 PE=1 SV=1 |
| E41L3 | Band 4.1-like protein 3 OS=Homo sapiens OX=9606 GN=EPB41L3 PE=1 SV=2 |
| NPA1P | Nucleolar pre-ribosomal-associated protein 1 OS=Homo sapiens OX=9606 GN=URB1 PE=1 SV=4 |
| PLIN2 | Perilipin-2 OS=Homo sapiens OX=9606 GN=PLIN2 PE=1 SV=2 |
| IPO8 | Importin-8 OS=Homo sapiens OX=9606 GN=IPO8 PE=1 SV=2 |
| RPAC1 | DNA-directed RNA polymerases I and III subunit RPAC1 OS=Homo sapiens OX=9606 GN=POLR1C PE=1 SV=1 |
| PKHG3 | Pleckstrin homology domain-containing family G member 3 OS=Homo sapiens OX=9606 GN=PLEKHG3 PE=1 SV=1 |
| VPS11 | Vacuolar protein sorting-associated protein 11 homolog OS=Homo sapiens OX=9606 GN=VPS11 PE=1 SV=1 |
| EMP2 | Epithelial membrane protein 2 OS=Homo sapiens OX=9606 GN=EMP2 PE=1 SV=1 |
| GTPBA | GTP-binding protein 10 OS=Homo sapiens OX=9606 GN=GTPBP10 PE=1 SV=1 |
| ANKE1 | Ankyrin repeat and EF-hand domain-containing protein 1 OS=Homo sapiens OX=9606 GN=ANKEF1 PE=2 SV=2 |
| NSMA3 | Sphingomyelin phosphodiesterase 4 OS=Homo sapiens OX=9606 GN=SMPD4 PE=1 SV=3 |
| CHM4B | Charged multivesicular body protein 4b OS=Homo sapiens OX=9606 GN=CHMP4B PE=1 SV=1 |
