# Supplementary material for: N6-methyladenosine reader protein YTHDC1 regulates influenza A virus NS segment splicing and replication
Source: PLoS Pathog. 2023 Apr 13;19(4):e1011305. doi: 10.1371/journal.ppat.1011305 (PMC10146569; doi:10.1371/journal.ppat.1011305)
Supplement: S2 Table — (DOCX) [file ppat.1011305.s008.docx]

**S2 Table. Analysis of GGAC motif in NS segment of H5, H7 and H9 IAVs.**

| NS without GGAC motif | | |
| --- | --- | --- |
| Strains | Year | Subtype |
| A/Chicken/Hong Kong/858.3/01 (H5N1) | 2002 | H5N1 |
| A/Canada goose/Sweden/V828/06(H5N1) | 2006 | H5N1 |
| A/Canada goose/Sweden/V978/06(H5N1) | 2006 | H5N1 |
| A/mute swan/Sweden/V827/06(H5N1) | 2006 | H5N1 |
| A/chicken/Nebraska/15-015085-1/2015(H5N2) | 2015 | H5N2 |
| A/chicken/Nebraska/15-016865-1/2015(H5N2) | 2015 | H5N2 |
| A/chicken/Nebraska/15-017897-1/2015(H5N2) | 2015 | H5N2 |
| A/chicken/Nebraska/15-017990-5/2015(H5N2) | 2015 | H5N2 |
| A/chicken/Nebraska/15-019197-1/2015(H5N2) | 2015 | H5N2 |
| A/environment/Nebraska/L3/2015(H5N2) | 2015 | H5N2 |
| A/environment/Nebraska/L4/2015(H5N2) | 2015 | H5N2 |
| A/environment/Nebraska/L5/2015(H5N2) | 2015 | H5N2 |
| A/environment/Nebraska/L6/2015(H5N2) | 2015 | H5N2 |
| A/environment/Nebraska/L7/2015(H5N2) | 2015 | H5N2 |
| A/environment/Nebraska/L8/2015(H5N2) | 2015 | H5N2 |
| A/environment/Nebraska/L9/2015(H5N2) | 2015 | H5N2 |
| A/chicken/Nebraska/15-015085-1/2015(H5N2) | 2015 | H5N2 |
| A/chicken/Nebraska/15-016865-1/2015(H5N2) | 2015 | H5N2 |
| A/chicken/Nebraska/15-017897-1/2015(H5N2) | 2015 | H5N2 |
| A/chicken/Nebraska/15-017990-5/2015(H5N2) | 2015 | H5N2 |
| A/chicken/Nebraska/15-019197-1/2015(H5N2) | 2015 | H5N2 |
| A/environment/Nebraska/L3/2015(H5N2) | 2015 | H5N2 |
| A/environment/Nebraska/L4/2015(H5N2) | 2015 | H5N2 |
| A/environment/Nebraska/L5/2015(H5N2) | 2015 | H5N2 |
| (A/environment/Nebraska/L6/2015(H5N2) | 2015 | H5N2 |
| A/environment/Nebraska/L7/2015(H5N2) | 2015 | H5N2 |
| A/environment/Nebraska/L8/2015(H5N2) | 2015 | H5N2 |
| A/environment/Nebraska/L9/2015(H5N2) | 2015 | H5N2 |
| A/turkey/Italy/16/2000(H7N1) | 2000 | H7N2 |
| A/Guinea_fowl/New_York/11646-3/2005(H7N2) | 2005 | H7N2 |
| A/guinea_fowl/New_York/143646-3/2005(H7N2) | 2005 | H7N2 |
| A/guinea_fowl/New_York/23164-3-05/2005(H7N2) | 2005 | H7N2 |
| A/Guinea_fowl/New_York/88291-9/2005(H7N2) | 2005 | H7N2 |
| A/Muscovy_duck/New_York/11646-4/2005(H7N2) | 2005 | H7N2 |
| A/muscovy_duck/New_York/23164-10/2005(H7N2) | 2005 | H7N2 |
| A/chicken/New_York/10508/2005(H7N2) | 2005 | H7N2 |
| A/chicken/New_York/11646-2/2005(H7N2) | 2005 | H7N2 |
| A/chicken/New_York/143646-2/2005(H7N2) | 2005 | H7N2 |
| A/chicken/New_York/16326-3/2005(H7N2) | 2005 | H7N2 |
| A/chicken/New_York/16326-4/2005(H7N2) | 2005 | H7N2 |
| A/chicken/New_York/16326-5/2005(H7N2) | 2005 | H7N2 |
| A/chicken/New_York/16330/2005(H7N2) | 2005 | H7N2 |
| A/chicken/New_York/19499/2005(H7N2) | 2005 | H7N2 |
| A/chicken/New_York/23164-4/2005(H7N2) | 2005 | H7N2 |
| A/chicken/New_York/23164-5/2005(H7N2) | 2005 | H7N2 |
| A/chicken/New_York/23164-6/2005(H7N2) | 2005 | H7N2 |
| A/chicken/New_York/23164-7/2005(H7N2) | 2005 | H7N2 |
| A/chicken/New_York/23164-8/2005(H7N2) | 2005 | H7N2 |
| A/chicken/New_York/23164-9/2005(H7N2) | 2005 | H7N2 |
| A/chicken/New_York/63806-11/2005(H7N2) | 2005 | H7N2 |
| A/chicken/New_York/63806-12/2005(H7N2) | 2005 | H7N2 |
| A/chicken/New_York/63806-7/2005(H7N2) | 2005 | H7N2 |
| A/chicken/New_York/63806-8/2005(H7N2) | 2005 | H7N2 |
| A/chicken/New_York/63806-11/2005(H7N2) | 2005 | H7N2 |
| A/chicken/New_York/63806-12/2005(H7N2) | 2005 | H7N2 |
| A/chicken/New_York/63806-8/2005(H7N2) | 2005 | H7N2 |
| A/chicken/New_York/88291-10/2005(H7N2) | 2005 | H7N2 |
| A/chicken/New_York/88291-11/2005(H7N2) | 2005 | H7N2 |
| A/chicken/New_York/88291-14/2005(H7N2) | 2005 | H7N2 |
| A/chicken/New_York/88291-15/2005(H7N2) | 2005 | H7N2 |
| A/chicken/New_York/88291-6/2005(H7N2) | 2005 | H7N2 |
| A/chicken/New_York/88291-8/2005(H7N2) | 2005 | H7N2 |
| A/chukar/New_York/11653-1/2005(H7N2) | 2005 | H7N2 |
| A/chukar/New_York/23164-13/2005(H7N2) | 2005 | H7N2 |
| A/duck/New_York/143646-5/2005(H7N2) | 2005 | H7N2 |
| A/duck/New_York/88291-12/2005(H7N2) | 2005 | H7N2 |
| A/duck/New_York/88291-13/2005(H7N2) | 2005 | H7N2 |
| A/environment/New_York/139052/2005(H7N2) | 2005 | H7N2 |
| A/environment/New_York/143646-1/2005(H7N2) | 2005 | H7N2 |
| A/environment/New_York/16326-1/2005(H7N2) | 2005 | H7N2 |
| A/environment/New_York/16326-2/2005(H7N2) | 2005 | H7N2 |
| A/environment/New_York/23164-1/2005(H7N2) | 2005 | H7N2 |
| A/environment/New_York/23164-2/2005(H7N2) | 2005 | H7N2 |
| A/environment/New_York/63806-1/2005(H7N2) | 2005 | H7N2 |
| A/environment/New_York/63806-2/2005(H7N2) | 2005 | H7N2 |
| A/environment/New_York/63806-3/2005(H7N2) | 2005 | H7N2 |
| A/environment/New_York/63806-4/2005(H7N2) | 2005 | H7N2 |
| A/environment/New_York/63806-5/2005(H7N2) | 2005 | H7N2 |
| A/environment/New_York/81928-2/2005(H7N2) | 2005 | H7N2 |
| A/environment/New_York/88291-1/2005(H7N2) | 2005 | H7N2 |
| A/environment/New_York/88291-2/2005(H7N2) | 2005 | H7N2 |
| A/environment/New_York/88291-3/2005(H7N2) | 2005 | H7N2 |
| A/environment/New_York/88291-4/2005(H7N2) | 2005 | H7N2 |
| A/environment/New_York/88291-5/2005(H7N2) | 2005 | H7N2 |
| A/pheasant/New_York/143646-4/2005(H7N2) | 2005 | H7N2 |
| A/quail/New_York/23164-12/2005(H7N2) | 2005 | H7N2 |
| A/quail/New_York/63806-13/2005(H7N2) | 2005 | H7N2 |
| A/turkey/New_York/23164-11/2005(H7N2) | 2005 | H7N2 |
| A/turkey/New_York/88291-7/2005(H7N2) | 2005 | H7N2 |
| A/unknown/New_York/11646-5/2005(H7N2) | 2005 | H7N2 |
| A/unknown/New_York/11646-6/2005(H7N2) | 2005 | H7N2 |
| A/unknown/New_York/11646-7/2005(H7N2) | 2005 | H7N2 |
| A/unknown/New_York/11646-8/2005(H7N2) | 2005 | H7N2 |
| A/unknown/New_York/11646-9/2005(H7N2) | 2005 | H7N2 |
| A/unknown/New_York/19501-5/2006(H7N2) | 2005 | H7N2 |
| A/unknown/New_York/88291-16/2005(H7N2) | 2005 | H7N2 |
| A/unknown/New_York/88291-17/2005(H7N2) | 2005 | H7N2 |
| A/Guinea_fowl/New_York/19495-6/2006(H7N2) | 2006 | H7N2 |
| A/guinea_fowl/New_York/19501-4/2006(H7N2) | 2006 | H7N2 |
| A/guinea_fowl/New_York/32084/2006(H7N2) | 2006 | H7N2 |
| A/guinea_fowl/New_York/8391-1/2006(H7N2) | 2006 | H7N2 |
| A/Muscovy_duck/New_York/19495-7/2006(H7N2) | 2006 | H7N2 |
| A/blue-winged_teal/Ohio/566/2006(H7N9) | 2006 | H7N2 |
| A/chicken/New_York/19495-2/2006(H7N2) | 2006 | H7N2 |
| A/chicken/New_York/19495-3/2006(H7N2) | 2006 | H7N2 |
| A/chicken/New_York/19495-4/2006(H7N2) | 2006 | H7N2 |
| A/chicken/New_York/19495-5/2006(H7N2) | 2006 | H7N2 |
| A/chicken/New_York/19499-1/2006(H7N2) | 2006 | H7N2 |
| A/chicken/New_York/29047-4/2006(H7N2) | 2006 | H7N2 |
| A/chicken/New_York/3181-5/2006(H7N2) | 2006 | H7N2 |
| A/chicken/New_York/46545-2/2006(H7N2) | 2006 | H7N2 |
| A/chicken/New_York/8391-2/2006(H7N2) | 2006 | H7N2 |
| A/chicken/New_York/46545-2/2006(H7N2) | 2006 | H7N2 |
| A/chicken/New_York/8391-2/2006(H7N2) | 2006 | H7N2 |
| A/environment/New_York/19495-1/2006(H7N2) | 2006 | H7N2 |
| A/environment/New_York/19501-1/2006(H7N2) | 2006 | H7N2 |
| A/environment/New_York/19501-2/2006(H7N2) | 2006 | H7N2 |
| A/environment/New_York/19501-3/2006(H7N2) | 2006 | H7N2 |
| A/environment/New_York/29047-1/2006(H7N2) | 2006 | H7N2 |
| A/environment/New_York/29047-2/2006(H7N2) | 2006 | H7N2 |
| A/environment/New_York/29047-3/2006(H7N2) | 2006 | H7N2 |
| A/environment/New_York/3181-1/2006(H7N2) | 2006 | H7N2 |
| A/environment/New_York/3181-2/2006(H7N2) | 2006 | H7N2 |
| A/environment/New_York/3181-3/2006(H7N2) | 2006 | H7N2 |
| A/environment/New_York/3181-4/2006(H7N2) | 2006 | H7N2 |
| A/environment/New_York/3185-1/2006(H7N2) | 2006 | H7N2 |
| A/environment/New_York/3185-2/2006(H7N2) | 2006 | H7N2 |
| A/environment/New_York/3185-3/2006(H7N2) | 2006 | H7N2 |
| A/environment/New_York/3185-4/2006(H7N2) | 2006 | H7N2 |
| A/environment/New_York/3185-5/2006(H7N2) | 2006 | H7N2 |
| A/environment/New_York/46545-1/2006(H7N2) | 2006 | H7N2 |
| A/feline/New_York/16-040082-1/2016(H7N2) | 2016 | H7N2 |
| A/feline/New_York/WVDL-14/2016(H7N2) | 2016 | H7N2 |
| A/feline/New_York/WVDL-16/2016(H7N2) | 2016 | H7N2 |
| A/feline/New_York/WVDL-20/2016(H7N2) | 2016 | H7N2 |
| A/feline/New_York/WVDL-3/2016(H7N2) | 2016 | H7N2 |
| A/feline/New_York/WVDL-9/2016(H7N2) | 2016 | H7N2 |
| A/wild_duck/Korea/MHC39-13/2011(H7N9) | 2011 | H7N9 |
| A/wild_duck/Korea/MHC39-26/2011(H7N9) | 2011 | H7N9 |
| A/wild_pigeon/Jiangsu/SD001/2013(H7N9) | 2013 | H7N9 |
| A/mallard_duck/Netherlands/1/2005(H9N2) | 2005 | H9N2 |
| A/chicken/Bangladesh/VP01/2006(H9N2) | 2006 | H9N2 |
| A/chicken/Hong_Kong/TC176/2006(H9N2) | 2006 | H9N2 |
| A/chicken/Shandong/LY/2006(H9N2) | 2006 | H9N2 |
| A/chicken/Hebei/0329/2007(H9N2) | 2007 | H9N2 |
| A/silkie_chicken/Hong_Kong/HH137/2008(H9N2) | 2008 | H9N2 |
| A/chicken/Jilin/3/2009(H9N2) | 2009 | H9N2 |
| A/ruddy_turnstone/New_Jersey/AI03-128/2003(H9N7) | 2003 | H9N7 |
