# Supplementary material for: N6-methyladenosine reader protein YTHDC1 regulates influenza A virus NS segment splicing and replication
Source: PLoS Pathog. 2023 Apr 13;19(4):e1011305. doi: 10.1371/journal.ppat.1011305 (PMC10146569; doi:10.1371/journal.ppat.1011305)
Supplement: S3 Table — (DOCX) [file ppat.1011305.s009.docx]

**S3 Table. Primers and probes**

| **Primers for qPCR** | |
| --- | --- |
| Name | Sequence |
| RT-NP-mRNA (RT) | CCAGATCGTTCGAGTCGTTTTTTTTTTTTTTTTTCTTTAATTGTC |
| RT-NP-cRNA  (RT) | GCTAGCTTCAGCTAGGCATCAGTAGAAACAAGGGTATTTTTCTTT |
| RT-NP-vRNA (RT) | GGCCGTCATGGTGGCGAATGAATGGACGGAGAACAAGGATTGC |
| NS1-F | GGAAGGGGCAGTACTCTCGG |
| NS1-R | TTTCTGCTTGGGTATGAGCA |
| NS1(stop)-F | GAAATGTCAAGGGACTGGT |
| NS1(stop)-R | AGCAATATTAGTCACTCCAG |
| NEP-F | CTGTGTCAAGCTTTCAGGAC |
| NEP-R | AGTCTCCCATCCTTATCACT |
| NP(mRNA)-F | CGATCGTGCCCTCCTTTG |
| NP(mRNA)-R | CCAGATCGTTCGAGTCGT |
| NP(cRNA)-F | CGATCGTGCCCTCCTTTG |
| NP(cRNA)-R | GCTAGCTTCAGCTAGGCATC |
| NP(vRNA)-F | GGCCGTCATGGTGGCGAAT |
| NP(vRNA)-R | CTCAATATGAGTGCAGACCGTGCT |
| YTHDC1-F | AACTGGTTTCTAAGCCACTGAGC |
| YTHDC1-R | GGAGGCACTACTTGATAGACGA |
| GAPDH-F | GCAAAGGCTGTGGGCAAGG |
| GAPDH-R | GGAGGAGTGGGTGTCGCTG |
| **Primers for amplification** | |
| YTHDC1-F | CGGAATTCATGGCGGCTGACAGTCG |
| YTHDC1-R | CCGCTCGAGTCTTCTATATCGACCTCTCTCCCCT |
| NS1-F | GCGAATTCAATGGATCCAAACACTGTGTCAAG |
| NS1-R | GCTCTAGAAACTTCTGACCTAATTGTTCCC |
| NS(45 stop)-F | ATGGATCCAAACACTGTGTGAAGCTTT |
| NS(45 stop)-R | AGAAAGCAATCTACCTGAAAGCTTCACACAG |
| NS(456 stop)-F | GTGATTTTTGACCGGCTGGAGTGACTAATA |
| NS(456 stop)-R | GTGAAAGCCCTTAGCAATATTAGTCACTCCAG |
| **RNA oligonucleotides for RNA pull-down** | |
| NS_WT(m6A)_ | CCUUCUCUUCCAGG(m^6^A)CAUAC |
| NS_WT_ | CCUUCUCUUCCAGGACAUAC |
| NS_A518C_ | CCUUCUCUUCCAGGCCAUAC |
